# Cigarette smoking, coffee consumption, alcohol intake, and clozapine metabolism: A Mendelian randomization study

**DOI:** 10.3389/fpsyt.2022.1002235

**Published:** 2022-09-29

**Authors:** Lingsi Zeng, Honggang Lv, Juan Li, Ranran Xue, Xia Liu, Cong Zhou, Hao Yu

**Affiliations:** ^1^Department of Psychiatry, Jining Medical University, Jining, China; ^2^Department of Psychiatry, Shandong Daizhuang Hospital, Jining, China

**Keywords:** Mendelian randomization, nicotine consumption, alcohol consumption, coffee consumption, clozapine concentration

## Abstract

**Background:**

Clozapine is an effective antipsychotic medication for patients with treatment-resistant schizophrenia. Previous studies revealed that smoking, alcohol intake, and coffee consumption altered the metabolism of clozapine. However, causal associations between substance use and clozapine levels were not sufficiently established.

**Methods:**

Several genome-wide association studies provided genetic tools for six measures of substance use, including age of smoking, cigarettes per day, smoking cessation, smoking initiation, coffee consumption, and alcohol consumption (GWASs). Utilizing the CLOZUK consortium’s dataset, their associations with clozapine and its metabolite concentrations were evaluated. All GWAS data were collected from the European population. Mendelian randomization (MR) estimations from each genetic test were combined using inverse variance weighted (IVW) meta-analysis in combination with complementing techniques (such as weighted median and MR Egger). We also analyze horizontal pleiotropy and heterogeneity using various sensitivity analyses.

**Results:**

Genetically predicted higher level of smoking initiation was significantly associated with reduced clozapine (β = –0.14, *P* = 4.53E-04) concentrations and norclozapine concentrations (β = –0.14, *P* = 3.33E-04), and increased coffee consumption was significantly associated with lower level of clozapine concentrations (β = –0.42, *P* = 1.70E-14), norclozapine concentrations (β = –0.27, *P* = 1.51E-07), and the metabolic ratio of clozapine to norclozapine (β = –0.15, *P* = 5.35E-07), survived after the Bonferroni correction (*P* = 0.05/6 = 0.008). In sensitivity analyses, the weighted median and MR Egger methods demonstrated directionally consistent effects. In addition, our sensitive test indicated no significant horizontal pleiotropy and heterogeneity (*P* > 0.05). However, other measures of substance use (age of initiation smoking, cigarettes per day, smoking cessation, and drinks per week) were not associated with clozapine metabolism.

**Conclusion:**

Our investigation revealed a correlation between greater smoking initiation and coffee consumption and reduced blood levels of clozapine and norclozapine. Providing clinicians with guidance on how to adjust clozapine levels for clozapine-treated patients.

## Introduction

Schizophrenia, which affects around 1% of the world’s population (1), is characterized by positive symptoms, negative symptoms, cognitive impairment and severe functional impairment. A third of patients suffer from treatment-resistant schizophrenia, which is characterized by substantial functional impairment and symptoms that do not respond sufficiently to at least two antipsychotics of first-line treatment (2). Clozapine is the sole antipsychotic indicated for treatment-resistant schizophrenia, and it is more successful than other antipsychotics at alleviating psychotic symptoms (3). Clozapine is only licensed as a third-line medication in the majority of developed nations due to its negative effects (4). Severe adverse medication events, such as seizures, tachycardia, sedation, weight gain, and hypersalivation, have been related with either clozapine dose or plasma concentration and are a major factor limiting clozapine’s usage (5). Conventionally, clinicians use clozapine concentration to alter dose for both therapeutic response and adverse medication responses (6). This is a crucial approach, as adverse effects are the leading cause of clozapine discontinuation (7). The variables that influence clozapine metabolism are not fully understood.

Previous research revealed that genetic and lifestyle variables may influence the metabolism of clozapine (5, 8–10). Genetic variations of the cytochrome P450 (CYP) and uridine diphosphate glycosyltransferase (UGT) families altered the metabolism of clozapine or its metabolites, according to a pharmacogenomic research (8). Patients with schizophrenia have elevated rates of drug abuse compared to the general population (7, 11). There is accumulating evidence that addicitive substances abuse plays a significant role in regulating clozapine metabolism (10). Several studies have indicated that smoking decreases blood clozapine concentrations (9, 10). Coffee containing caffeine raised the mean blood concentration of clozapine by 20–26% and lowered the ratio of N-desmethylclozapine/clozapine by 9–13% (9). Alcohol intake may influence the therapeutic effectiveness of clozapine by altering its absorption and metabolism (12, 13). Consequently, it is essential to determine if addicitive substances abuse has a clinically relevant impact on clozapine metabolism.

Although multiple studies have identified a correlation between drug usage and clozapine levels, their causal relationship is unclear due to confounding variables. A randomized controlled trial study is the best method for verifying causality, but it is lengthy, expensive, and fraught with problems, so a successful approach is to use genetic variants as exposure indicators that are not subject to the influences that compromise conventional study designs, an approach known as Mendelian randomization (MR) (14, 15). The benefit of MR investigations is based on three assumptions, utilizes the random assignment of alleles during meiotic cell division and conception as the foundation of a natural experiment (14, 16). Using data from existing databases, the MR analysis avoided the impact of reverse causality confounders. The data utilized for smoking, coffee, and alcohol came from a verified GWAS database. These advantages possessed by Mendelian randomization above prompted the continuation of our study.

Herein, we employed the two-sample MR method to assess the effect of smoking, coffee drinking, or alcohol consumption on clozapine metabolism in order to determine if substance use in typically ingested quantities effects clozapine concentrations in individuals with schizophrenia.

## Materials and methods

### Study design

**FIGURE 1:**
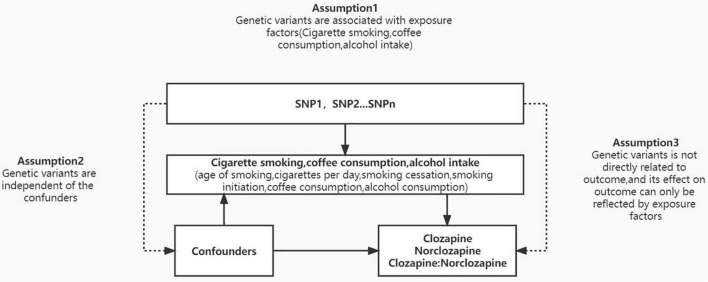
Mendelian randomization (MR) model. We conducted MR analysis based on three hypotheses: (1) the instrumental variables for each measure of substance use are associated with exposure factors; (2) the instrumental variables are not associated with any confounders; and (3) the instrumental variables are only related with clozapine metabolism through substance use and not through any other causal pathway. According to MR assumptions, solid lines are hypothesized to exist whereas dashed lines are theorized to be insignificant.

To determine the causal relationship between substance abuse and clozapine metabolite plasma concentrations, we utilized an MR research design. It uses genetic variation as a tool variable to derive the causality of outcome and exposure, and is an effective method used to conduct causal inference, and can effectively avoid the confounding bias in traditional epidemiological studies. We selected nicotine consumption-related (include age of smoking, cigarettes per day, smoking cessation, smoking initiation), genetic variation related to coffee consumption and alcohol consumption as instrumental variables related to exposure factors, clozapine concentration, norclozapine and the ratio of the two as outcome variables. Based on this basis, we proposed the following three hypotheses (Figure 1): (1) The SNPs that were used as instrumental variables (IVs) for substance use measures were linked to exposure factors; (2) the IVs were not confounded by confounding variables; and (3) the IVs are only linked with the clozapine metabolism via the substance use measures but not through any other causal pathway. The expected connections between substance use indicators and clozapine metabolism are linear, not statistical interactions. To obtain a comprehensive and conclusive causal relationship between nicotine, coffee consumption, and alcohol consumption with clozapine and its metabolites, we selected the most extensive GWAS database available to date.

### Data source

#### Exposures: Smoking, alcohol consumption, and coffee consumption

Age of initiation smoking, cigarettes per day, smoking cessation, smoking initiation, coffee consumption, and drinks per week were selected as the six exposure variables of substance use relevant to this study (5, 17, 18). A large-scale meta-analysis of 33 GWAS datasets was utilized to extract data about the association between genome-wide associated SNPs and smoking and alcohol consumption. For smoking, we focused on four phenotypes: age of initiation of regular smoking (age of smoking, *N* = 341,427), cigarettes per day (CPD, *N* = 337,334), smoking initiation (showing an individual had ever smoked regularly, *N* = 1,232,091), and smoking cessation (*N* = 547,475). For alcohol consumption, the number of drinks per week phenotype (*N* = 941,280) was utilized. A genome-wide meta-analysis (*N* = 91,462) yielded SNPs that were related with coffee consumption across the genome.

#### Outcomes: Clozapine metabolite plasma concentrations

Summary data for the associations between the selected instrumental variables and plasma concentrations of clozapine metabolite were extracted from the GWAS meta-analysis published by CLOZUK study, consisting of 2,989 individuals of European ancestry (8). Summary statistics from the CLOZUK GWASs are available for download at http://walters.psycm.cf.ac.uk/. In the present study, we used three metabolic outcome variables as outcomes, including the plasma concentrations of clozapine and norclozapine and the ratio of clozapine to norclozapine (metabolic ratio). The CLOZUK study conducted GWAS analysis through GCTA (Genome-wide Complex Trait Analysis) software (19), which could control for family and population structure, and sex. Table 1 summarizes the basic features of the GWAS samples. More information about the GWAS samples was provided in the original papers (5, 8, 17, 18).

**TABLE 1:** Description of GWAS summary statistics used for each phenotype.

Phenotype	References	Consortium	Sample size	Population
Clozapine	Pardiñas et al. (8)	CLOZUK2	2,989 participants	European
Norclozapine	Pardiñas et al. (8)	CLOZUK2	2,989 participants	European
Metabolic ratio	Pardiñas et al. (8)	CLOZUK2	2,989 participants	European
Age of smoking	Liu et al. (5)	GSCAN	341,427 participants	European
Cigarettes per day	Liu et al. (5)	GSCAN	337,334 participants	European
Smoking cessation	Liu et al. (5)	GSCAN	547,475 participants	European
Smoking initiation	Liu et al. (5)	GSCAN	1,232,091 participants	European
Coffee consumption	Coffee and Caffeine Genetics Consortium et al. (17)	N.A	91,462participants	European, African American
Alcohol consumption	Liu et al. (5)	GSCAN	941,280 participants	European

We searched PubMed for GWASs of 6 exposures and identified genetic variants with genome-wide significant (*P* < 5 × 10^–8^) associations for reference levels of age of smoking Liu et al. (5), cigarettes per day, smoking cessation, smoking initiation, alcohol consumption Liu et al. (5), and coffee consumption Coffee and Caffeine Genetics Consortium et al. (17). Then, we extracted the associations between genetic instrument and clozapine, Pardiñas et al. (8), norclozapine, metabolic ratio, from the GWAS data of Psychiatric Genomics Consortium (PGC, http://www.med.unc.edu/pgc/).

### Selection of instrumental variables

We used linkage disequilibrium (LD) independent SNPs linked with the drug use indicator at *P* < 5 × 10^–8^ as IVs in our MR analysis. When the SNPs were significantly linked, we chose the SNP that had the lowest *P*-value in connection to substance use. Totally, 9 SNPs were identified for smoking age, 72 for cigarettes per day, 15 for smoking start, 200 for smoking cessation, 72 for drinks per week, and 8 for coffee intake (Supplementary Tables 1–6). In order to measure the efficacy of the chosen IVs, we also calculated the *F*-statistics, which are normally suggested for MR analysis. The differences present in each genetic instrument used in this study are listed in Supplementary Tables 1–6.

### Mendelian randomization analyses

To harmonize the exposure factors and outcome summary statistics from the GWAS, we matched the summary statistics on the reference allele and excluded palindromic variants with uncertain allele frequency from the MR analysis (20). Using the inverse-variance weighted (IVW) method, we combined SNP-specific causal estimates for clozapine metabolism (21). The significant threshold for multiple tests was *P* < 0.003 (0.05/18 = 0.003) when the Bonferroni method was applied, since we conducted MR analyses of six addictive substances use indices and three clozapine metabolism indicators. Genetic instruments were examined for heterogeneity and directional pleiotropy using a weighted median function and MR-Egger regression (22, 23). To evaluate the validity of statistically significant results, the Cochran Q statistic test was used to identify heterogeneous results (23). We switched from the inverse variance-weighted fixed model to the random-effects model if Cochran’s Q indicated heterogeneity and pleiotropy (*P* < 0.05) (24). We carried out a leave-one-out study in which one SNP was eliminated at a time in order to evaluate the influence of marginal and/or pleiotropic SNPs (25). We used the MR-Pleiotropy RESidual Sum and Outlier (MR-PRESSO) program to identify SNP outliers (26). In order to decrease any pleiotropic effects, we excluded these outliers and reanalyzed the associations without them. Genetically predicted increases in substance use are shown as effect estimates with 95% confidence intervals (CIs). R (version 3.4.0) and the TwoSampleMR package were used to conduct all statistical analyses (27).

## Results

**FIGURE 2:**
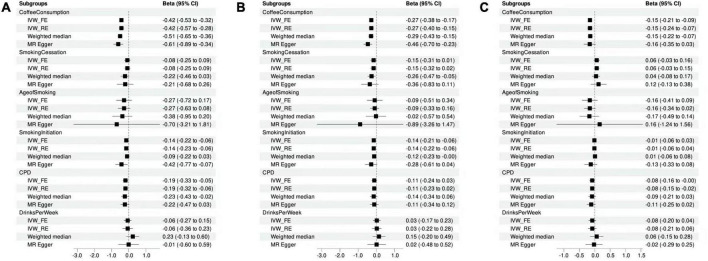
Mendelian randomization analysis showing the effects of cigarette smoking, coffee consumption, alcohol intake, and clozapine metabolism. **(A)** MR analyses of the measures of substance use and clozapine concentrations. **(B)** MR analyses of the measures of substance use and norclozapine concentrations. **(C)** MR analyses of the measures of substance use and the ratio of clozapine to norclozapine.

The number of IVs involved in this study varied from 8 to 200. In the selection phase of the instrumental variables, we removed some outliers and calculated the F statistics and variance for all SNP. The minimum F statistic in the last selected SNP is 9, indicating that there is no weak tool variable in this MR analysis, and the overall association of the IVW test is shown in Figure 2.

### Associations of cigarette smoking, coffee consumption, alcohol intake with clozapine concentration

Using MR-PRESSO method, we detected and removed two SNP outliers (rs17177078 and rs2472297) for the phenotype of drinking per week. After removing the outliers, we found significant casual association for smoking initiation (β = –0.14, *P* = 4.53E-04) and coffee consumption (β = –0.42, *P* = 1.70E-14) with the concentration of clozapine (Figure 2A and Supplementary Table 8), surviving after Bonferroni correction. The results of sensitivity analysis using weighted median and MR Egger analysis for the effects of smoking initiation and coffee consumption on the concentration of clozapine were comparable (Supplementary Table 8). Modified Q statistics indicated that instrument SNP effects were not significantly heterogeneous (*P* > 0.05; Supplementary Table 7). It was also shown that there was no horizontal pleiotropy by the MR-Egger intercept test (intercept = –0.01; SE, 0.01; *P* = 0.12; Supplementary Table 8). No one SNP was significantly linked to smoking initiation and coffee consumption as a result of our leaving-one-out analysis (Supplementary Figures 1, 2).

Moreover, cigarettes per day showed suggestive association with the concentration of clozapine (β = –0.19, *P* = 8.52E-03; Figure 2A). However, no significant results were found for age of smoking (β = –0.27, *P* = 0.23), smoking cessation (β = –0.08, *P* = 0.35), and alcohol consumption (β = –0.06, *P* = 0.57) with clozapine concentration (Supplementary Table 8).

### Associations of cigarette smoking, coffee consumption, alcohol intake with norclozapine concentration

Using MR-PRESSO method, we detected and removed one SNP outliers (rs2472297) for the phenotype of drinking per week. Then, we detected the significantly causal relationship of smoking initiation (β = –0.14, *P* = 3.33E-04) and coffee consumption (β = –0.27, *P* = 1.51E-07; Figure2B) with norclozapine concentration. Sensitivity analysis yielded similar results for causal effects of smoking initiation (Weighted median: β = –0.12, *P* = 0.05; MR Egger: β = –0.28, *P* = 0.09) and coffee consumption (Weighted median: β = –0.29, *P* = 6.27E-05; MR Egger: β = –0.46, *P* = 7.70E-03) on the concentration of norclozapine. No horizontal pleiotropy was observed (Supplementary Table 8). Additionally, no horizontal pleiotropy was detected by the MR-Egger intercept test (intercept = –0.01; SE, 0.01; *P* = 0.12; Supplementary Table 8). No one SNP was significantly linked to smoking initiation and coffee consumption as a result of our leaving-one-out analysis (Supplementary Figures 3, 4). No significant results were found for age of smoking (β = –0.09, *P* = 0.69), cigarettes per day (β = –0.11, *P* = 0.11), smoking cessation (β = –0.15, *P* = 0.06), and alcohol consumption (β = 0.03, *P* = 0.79) with clozapine concentration (Supplementary Table 8).

### Associations of cigarette smoking, coffee consumption, alcohol intake with metabolic ratio

We find no SNP outliers for the metabolic ratio of clozapine and norclozapine concentrations using the MR-PRESSO test. Our results showed that coffee consumption had a causal relationship with metabolic ratio of clozapine and norclozapine concentration (β = –0.15, *P* = 5.35E-07). Further, similar patterns of effects were observed when sensitivity analyses were conducted using weighted median and MR Egger analyses (Figure 2C). However, we found significant heterogeneity (*P* < 0.05), and changed from the inverse variance-weighted fixed model to the random-effects model. The coffee consumption was still significantly associated with metabolic ratio of clozapine and norclozapine concentration (β = –0.15, *P* = 2.65E-04), surviving after Bonferroni correction. Additionally, results from MR-Egger intercept tests showed no evidence of horizontal pleiotropy (intercept = –0.01; SE, 0.01; *P* = 0.12; Supplementary Table 8). Our leaving-one-out study revealed that no single SNP significantly affected the amount of coffee consumption (Supplementary Figure 5).

Moreover, age of smoking (β = –0.16, *P* = 0.21), cigarettes per day (β = –0.08, *P* = 0.05), smoking cessation (β = 0.06, *P* = 0.18), smoking initiation (β = –0.01, *P* = 0.61), and alcohol consumption (β = –0.08, *P* = 0.20) indicated no significant association with the metabolic ratio of clozapine and norclozapine concentration (Supplementary Table 8).

## Discussion

We sought to reliably quantify the effect of substance use on the concentrations of clozapine and its metabolite. To do this, we used the MR approach and found additional evidence that smoking initiation and coffee consumption were likely to reduce the concentration of clozapine or its metabolites. These findings corroborate many previous prospective observational studies that identified smoking and coffee intake to be at reduced concentration of clozapine and its metabolites. Given each SNP is allocated randomly at the time of conception, the MR results should be unconfounded by other factors for clozapine concentration, and therefore they can be thought of as approximately analogous to a series of natural randomized trials evaluating the effect of substance use on the concentration of clozapine. In contrast, we found little evidence that other factors, such as age of smoking, cigarettes per day, smoking cessation, and alcohol consumption were associated with clozapine metabolism. Our discovery is timely in that it raises awareness of the possible negative effects of smoking and coffee intake on focus while also offering more reliable scientific support for the treatment.

Smoking behavior and coffee consumption have been shown to play a key role in clozapine metabolism in previous studies (10, 28–30). Although a considerable number of studies have examined the influence of smoking behavior and coffee intake on clozapine concentration, but previous studies yielded conflicting results. For example, a recent meta-analysis comprising of 7,125 individuals found that clozapine blood levels are significantly lower in smokers compared with non-smokers (10). However, a prospective observational study indicated that the plasma concentrations of clozapine or norclozapine did not change significantly after the smoking ban for patients taking clozapine at inpatient hospital sites (28). According to a number of studies, coffee consumption slows down the metabolism of clozapine and raises clozapine levels in the body (29, 30). However, a clinical trial found that the effect of instant coffee drinking on serum clozapine concentrations is of minor clinical relevance in most of the patients (9).

In our MR study, we observed significant genetic evidence that smoking initiation and coffee consumption were associated with decreased concentration of clozapine and norclozapine, which enhances the evidence to establish a negative association between smoking and coffee with clozapine metabolism. Previously, it has been difficult to ascertain if there is a causal association between addictive substance usage and clozapine concentration. In observational studies, residual confounding may affect the results even after adjusting for confounding factors. As a result, determining causation has always been difficult, since randomized controlled studies could expose participants to adverse conditions. Notably, an MR study can provide important data on causation, which is essential to the public’s health and offers information for recommendations. Our study had a larger sample size and more statistical power than earlier observational studies. These noteworthy results from MR studies and conventional observational research demonstrate the causative relationships between smoking and coffee consumption and clozapine levels.

### Mechanisms of association

Our findings on the effects of smoking and coffee intake on clozapine levels are compatible with known biology. Clozapine is metabolized by CYP1A2 and CYP3A4 and partially also by CYP2C19. Inductors of these enzymes reduce while inhibitors raise clozapine levels. Clozapine and caffeine are metabolized largely by CYP1A2 enzyme. Polycyclic aromatic hydrocarbons in cigarettes are inductors of CYP1A2. Therefore, individuals treated with clozapine need to be more strictly watched if they discontinue smoking or coffee intake, since their plasma levels might quickly rise as the CYP1A2 induction declines. Therefore, individuals treated with clozapine need to be more strictly watched if they discontinue smoking or coffee intake, since their plasma levels can rapidly rise when the CYP1A2 induction declines. Because the smokers who were treated with clozapine were reported to suffer from substantial central nervous side effects after smoking cessation (31), it is vital to manage the clozapine dosage carefully when smokers cease smoking or lower the amount of smoking. In addition to studying the role of drug-metabolizing enzymes in antipsychotic therapy, more and more researchers have recently improved the safety and efficacy of drug therapy by combining new pharmacogenomic findings and therapeutic drug monitoring (TDM) (32). The role of TDM of plasma concentrations and pharmacogenetic testing is highlighted in the latest version of the GMT panel consensus guidelines in guiding drug therapy in clinical practice (33, 34). There is growing evidence that it is expected to adjust patient antipsychotic medication dosage through the measurement and interpretation of drug concentrations and when combined with a detailed understanding of the genetic patient background (35, 36). Evidence-based guidelines for selective serotonin reuptake inhibitor (SSRI) vs. tricyclic antidepressive agents (TCA) published by the Clinical Pharmacogenetics Implementation Consortium also give us some implications that pharmacogenetic testing and interpreting genotyping results can be used to optimize drug therapy. Our findings offer the basis for possible clinical uses of as a therapeutic monitoring for the management of individuals with treatment-resistant schizophrenia.

Pharmacogenomics study shown that genetic variants in UGT enzymes, which were responsible for nicotine glucuronidation, were also involved in clozapine metabolism (8). Previous caffeine clearance tests, an index of CYP1A2 activity, indicated that caffeine metabolism was correlated with clozapine clearance (37).

### Limitations

The present study’s strengths were the MR design and the utilization of summary-level data from the biggest GWASs to date. Under MR assumptions, this approach minimized bias owing to reverse causality and confounding. Moreover, the consistency of results from other sensitivity studies, such as the use of weighted mean, weighted median, and MR-egger techniques, indicates the robustness of our findings. However, there are numerous constraints to consider. Although our work utilized MR techniques to simplify the research process and eliminate the impact of confounding variables and reverse causality, it has limitations. First, the MR analysis instrument is incapable of completely avoiding horizontal pleiotropic effects, such as confounding variables. Second, based on two-sample data statistics, the causal association between coffee, smoking, and clozapine is ambiguous and requires more clinical research. This study can only examine causation, which is not clear enough regarding coffee consumption and smoking to provide a reliable reference, such as the amount of coffee or smoke that does not alter clozapine concentration. Due to a degree of chromosomal variances between ethnicities, it is questionable if these findings can be applied merely internationally to our European and African American participants. Using a combination of MR and case-control research, we may be able to comprehensively gather genome-wide comparison analyses between various ethnic groups, therefore resolving our existing uncertainties. The discovery of new genetic loci governing the connection between coffee consumption and clozapine concentration may give direction for future clinical marketing. Third, for the equally popular drink, tea, but we did not include the tea or benzodiazepine in the study, based on some of the following considerations. For example, but the chemicals of tea are too complex. Although many people are studying tea polyphenols now, we cannot guarantee that the amount of various chemicals and tea polyphenols are equal in each tea sample. Moreover, there are many kinds of teas, and since the chemical components they contain are not completely clear, less information exists on the pharmacokinetic effects of the chemical components of antipsychotic drugs, and there is no clear evidence on which biological mechanism is involved in the metabolism of antipsychotic drugs. Therefore, in order to ensure the reliability of the research results, tea was not included in the selection scope. But tea is always worth careful exploration in the study of psychiatric pharmacogenomics.

To our knowledge, this is the first study using MR approach to investigate the potentially causal relationship of addictive substances on clozapine metabolism. In summary, our findings supported potential associations between smoking initiation and coffee consumption and clozapine metabolism. Further investigations in understanding the underlying mechanisms of smoking and coffee intake in the metabolism of clozapine are required.

## Data availability statement

The original contributions presented in this study are included in the article/Supplementary material, further inquiries can be directed to the corresponding author.

## Author contributions

LZ and HY designed the study, contributed to analysis and interpretation of data, and wrote the first draft of the manuscript. LZ, HL, JL, XL, RX, CZ, and HY did the statistical analyses and prepared the table and figures. HY provided further data interpretation. All authors contributed to drafting the work and revising it critically for important intellectual content and made substantial contributions to the concept and design of the study and acquisition, analysis, and interpretation of data.
