# Supplementary material for: Cigarette smoking, coffee consumption, alcohol intake, and clozapine metabolism: A Mendelian randomization study
Source: Front Psychiatry. 2022 Sep 29;13:1002235. doi: 10.3389/fpsyt.2022.1002235 (PMC9559606; doi:10.3389/fpsyt.2022.1002235)

**Supplementary Table 1: Characteristics of genetic instruments associated with age of smoking**

| SNP | CHR | BP | A1 | A2 | FRQ | beta | se | P | F |
| --- | --- | --- | --- | --- | --- | --- | --- | --- | --- |
| rs72853300 | 2 | 145638766 | T | C | 0.153 | 0.019 | 0.003 | 1.75E-08 | 31.80 |
| rs12611472 | 2 | 225353649 | C | T | 0.297 | 0.018 | 0.003 | 3.48E-12 | 48.40 |
| rs7559982 | 2 | 63622309 | A | T | 0.564 | -0.017 | 0.002 | 1.67E-12 | 49.80 |
| rs11915747 | 3 | 85699040 | G | C | 0.354 | 0.020 | 0.003 | 1.57E-15 | 63.50 |
| rs13136239 | 4 | 140908755 | A | G | 0.342 | 0.015 | 0.003 | 6.29E-09 | 33.70 |
| rs2471711 | 4 | 28589079 | T | C | 0.152 | -0.019 | 0.003 | 1.19E-08 | 32.50 |
| rs624833 | 4 | 2881256 | G | T | 0.302 | 0.016 | 0.003 | 2.36E-09 | 35.70 |
| rs7682598 | 4 | 68000888 | G | A | 0.771 | 0.017 | 0.003 | 2.09E-09 | 35.90 |
| rs1403174 | 7 | 2032865 | T | A | 0.579 | 0.016 | 0.002 | 2.5E-10 | 40.00 |
| rs11780471 | 8 | 27344719 | A | G | 0.0601 | 0.033 | 0.005 | 9.44E-11 | 41.90 |

SNP, single nucleotide polymorphism; A1, effect allele; A2, other allele; FRQ, effect allele frequency; SE, standard error;

**Supplementary Table 2: Characteristics of genetic instruments associated with cigarettes per day**

| SNP | CHR | BP | A1 | A2 | FRQ | beta | se | P | F |
| --- | --- | --- | --- | --- | --- | --- | --- | --- | --- |
| rs11264100 | 1 | 35591626 | G | A | 0.876 | -0.022 | 0.004 | 2.22E-09 | 35.80 |
| rs2072659 | 1 | 154548521 | G | C | 0.0986 | -0.030 | 0.004 | 2.51E-13 | 53.60 |
| rs34973462 | 1 | 175993820 | T | C | 0.334 | 0.015 | 0.003 | 5.85E-09 | 33.90 |
| rs7599488 | 2 | 60718347 | T | C | 0.437 | 0.014 | 0.002 | 8.95E-09 | 33.10 |
| rs78408772 | 2 | 62710608 | T | C | 0.102 | -0.022 | 0.004 | 4.51E-08 | 29.90 |
| rs10204824 | 2 | 148372720 | G | A | 0.639 | -0.018 | 0.003 | 1.35E-12 | 50.30 |
| rs2084533 | 3 | 16872929 | T | C | 0.321 | 0.016 | 0.003 | 6.53E-10 | 38.20 |
| rs7431710 | 3 | 48935583 | A | G | 0.654 | -0.018 | 0.003 | 1.04E-12 | 50.80 |
| rs2236951 | 3 | 50421081 | C | T | 0.2 | -0.017 | 0.003 | 1.59E-08 | 31.90 |
| rs699165 | 3 | 136224697 | G | A | 0.745 | 0.016 | 0.003 | 8.09E-09 | 33.30 |
| rs28813180 | 3 | 158083918 | A | G | 0.498 | -0.015 | 0.002 | 1.95E-10 | 40.50 |
| rs1024323 | 4 | 3006043 | T | C | 0.382 | -0.014 | 0.003 | 8.66E-09 | 33.10 |
| rs11940255 | 4 | 67086288 | A | G | 0.717 | -0.017 | 0.003 | 2.2E-10 | 40.30 |
| rs10454798 | 4 | 67980830 | T | G | 0.253 | 0.016 | 0.003 | 1.53E-08 | 32.00 |
| rs7766641 | 6 | 26184102 | A | G | 0.272 | -0.017 | 0.003 | 2.91E-10 | 39.70 |
| rs215600 | 7 | 32333642 | A | G | 0.645 | -0.024 | 0.003 | 4.02E-21 | 89.00 |
| rs62447179 | 7 | 50339609 | A | G | 0.298 | -0.015 | 0.003 | 9.68E-09 | 32.90 |
| rs2741351 | 8 | 27418040 | C | A | 0.826 | 0.018 | 0.003 | 8.8E-09 | 33.10 |
| rs73229090 | 8 | 27442127 | A | C | 0.112 | 0.026 | 0.004 | 1.14E-11 | 46.10 |
| rs13253502 | 8 | 42442018 | A | G | 0.407 | -0.014 | 0.002 | 2.31E-08 | 31.20 |
| rs4236926 | 8 | 42578059 | G | T | 0.766 | 0.034 | 0.003 | 7.66E-33 | 142.00 |
| rs790564 | 8 | 64604218 | C | A | 0.729 | -0.018 | 0.003 | 1.24E-10 | 41.40 |
| rs75596189 | 9 | 136468701 | T | C | 0.112 | 0.036 | 0.004 | 1.84E-20 | 86.00 |
| rs3025383 | 9 | 136502369 | C | T | 0.187 | -0.031 | 0.003 | 9.78E-24 | 101.00 |
| rs7951365 | 11 | 16377044 | C | T | 0.31 | 0.018 | 0.003 | 1.53E-11 | 45.50 |
| rs10742683 | 11 | 43667625 | A | G | 0.415 | -0.013 | 0.002 | 4.83E-08 | 29.80 |
| rs113001570 | 11 | 46737412 | T | A | 0.0667 | 0.030 | 0.005 | 1.04E-09 | 37.30 |
| rs7125588 | 11 | 113436072 | G | A | 0.429 | -0.017 | 0.002 | 6.5E-12 | 47.20 |
| rs11846838 | 14 | 104184737 | A | G | 0.327 | 0.015 | 0.003 | 5.03E-09 | 34.20 |
| rs1115019 | 15 | 57141231 | C | T | 0.79 | -0.018 | 0.003 | 2.27E-09 | 35.70 |
| rs632811 | 15 | 59155050 | G | A | 0.33 | -0.018 | 0.003 | 1.67E-10 | 40.80 |
| rs4886550 | 15 | 78243579 | G | A | 0.288 | -0.020 | 0.003 | 4.58E-09 | 34.40 |
| rs12438181 | 15 | 78812098 | A | G | 0.218 | -0.019 | 0.003 | 4.97E-10 | 38.70 |
| rs10519203 | 15 | 78814046 | A | G | 0.655 | -0.094 | 0.003 | 3.1E-286 | 1309.99 |
| rs28438420 | 15 | 78836288 | T | A | 0.554 | 0.018 | 0.002 | 1.25E-12 | 50.40 |
| rs72740955 | 15 | 78849779 | T | C | 0.337 | 0.032 | 0.003 | 2.42E-34 | 149.00 |
| rs146009840 | 15 | 78906177 | T | A | 0.335 | 0.022 | 0.003 | 2E-17 | 72.10 |
| rs28681284 | 15 | 78908565 | T | C | 0.21 | -0.049 | 0.003 | 2.1E-58 | 260.00 |
| rs8040868 | 15 | 78911181 | C | T | 0.4 | 0.016 | 0.003 | 1.79E-10 | 40.70 |
| rs3743063 | 15 | 79065171 | C | A | 0.562 | -0.017 | 0.002 | 1.53E-11 | 45.50 |
| rs182317 | 15 | 89943601 | T | G | 0.355 | -0.016 | 0.003 | 1.31E-09 | 36.80 |
| rs1592485 | 16 | 52093549 | A | C | 0.611 | -0.016 | 0.003 | 1.11E-10 | 41.60 |
| rs12924872 | 16 | 69552215 | T | C | 0.463 | -0.013 | 0.002 | 4.39E-08 | 30.00 |
| rs258321 | 16 | 89756473 | G | A | 0.429 | 0.016 | 0.002 | 1.53E-10 | 41.00 |
| rs4144686 | 18 | 53251725 | A | G | 0.167 | -0.019 | 0.003 | 1.35E-08 | 32.30 |
| rs4485470 | 18 | 62125063 | A | G | 0.592 | -0.015 | 0.002 | 7.05E-10 | 38.00 |
| rs59208569 | 19 | 4044424 | C | G | 0.829 | 0.020 | 0.003 | 2.45E-10 | 40.10 |
| rs143200968 | 19 | 41338847 | C | G | 0.0251 | -0.086 | 0.008 | 6.97E-28 | 120.00 |
| rs56113850 | 19 | 41353107 | C | T | 0.555 | 0.052 | 0.002 | 4E-99 | 447.00 |
| rs8192726 | 19 | 41354496 | A | C | 0.0679 | -0.039 | 0.005 | 8.35E-16 | 64.80 |
| rs117824460 | 19 | 41371480 | G | A | 0.026 | -0.095 | 0.008 | 7.66E-35 | 152.00 |
| rs6078373 | 20 | 11863500 | A | G | 0.402 | 0.016 | 0.002 | 9.4E-11 | 41.90 |
| rs1737894 | 20 | 31054702 | G | C | 0.408 | 0.017 | 0.002 | 9.9E-12 | 46.30 |
| rs2273500 | 20 | 61986949 | C | T | 0.147 | 0.036 | 0.003 | 3.49E-26 | 112.00 |
| rs7281463 | 21 | 40520783 | C | A | 0.413 | 0.014 | 0.002 | 3.15E-08 | 30.60 |

SNP, single nucleotide polymorphism; A1, effect allele; A2, other allele; FRQ, effect allele frequency; SE, standard error;

**Supplementary Table 3: Characteristics of genetic instruments associated with smoking cessation.**

| SNP | CHR | BP | A1 | A2 | FRQ | beta | se | P | F |
| --- | --- | --- | --- | --- | --- | --- | --- | --- | --- |
| rs112187834 | 2 | 23953454 | A | A | 0.14 | 0.033 | 0.006 | 2.81E-09 | 35.30 |
| rs7617480 | 3 | 49210732 | C | C | 0.773 | -0.033 | 0.005 | 1.68E-12 | 49.80 |
| rs12203592 | 6 | 396321 | T | T | 0.176 | -0.029 | 0.005 | 1.21E-08 | 32.50 |
| rs707968 | 6 | 35058117 | G | G | 0.681 | 0.023 | 0.004 | 2.76E-08 | 30.90 |
| rs7778443 | 7 | 32314690 | C | C | 0.618 | -0.023 | 0.004 | 1.04E-08 | 32.80 |
| rs1565735 | 8 | 27426077 | A | A | 0.199 | -0.035 | 0.005 | 1.54E-12 | 50.00 |
| rs60749569 | 8 | 42602668 | T | T | 0.0796 | -0.040 | 0.007 | 2.68E-08 | 30.90 |
| rs12378015 | 9 | 127917257 | A | A | 0.3 | -0.028 | 0.004 | 8.31E-11 | 42.20 |
| rs9409844 | 9 | 136461851 | A | A | 0.04523 | -0.059 | 0.009 | 4.37E-10 | 38.90 |
| rs3025327 | 9 | 136467344 | C | C | 0.107 | 0.079 | 0.006 | 1.19E-35 | 155.00 |
| rs10821523 | 9 | 136473572 | C | C | 0.535529 | 0.026 | 0.004 | 2.28E-11 | 44.70 |
| rs1611124 | 9 | 136509275 | T | T | 0.067749 | -0.045 | 0.008 | 5.26E-09 | 34.10 |
| rs7109376 | 11 | 16372431 | A | A | 0.279 | 0.028 | 0.004 | 1.14E-10 | 41.60 |
| rs591143 | 15 | 47647755 | T | T | 0.592 | -0.024 | 0.004 | 1.14E-09 | 37.10 |
| rs3866543 | 15 | 76629609 | G | G | 0.523 | 0.022 | 0.004 | 1.35E-08 | 32.30 |
| rs518425 | 15 | 78883813 | G | G | 0.285 | -0.031 | 0.004 | 1.72E-12 | 49.80 |
| rs145580088 | 19 | 41342842 | G | G | 0.024 | 0.091 | 0.013 | 9.48E-13 | 50.90 |
| rs56113850 | 19 | 41353107 | C | C | 0.567 | -0.058 | 0.004 | 1.61E-48 | 214.00 |
| rs117824460 | 19 | 41371480 | G | G | 0.0265 | 0.086 | 0.012 | 1.09E-12 | 50.70 |
| rs59586387 | 19 | 41375030 | G | G | 0.0679 | 0.051 | 0.008 | 3.37E-11 | 43.90 |
| rs6011779 | 20 | 61984317 | T | T | 0.806 | -0.050 | 0.005 | 9.89E-24 | 101.00 |
| rs4809543 | 20 | 61986950 | A | A | 0.075645 | 0.044 | 0.007 | 2.4E-09 | 35.60 |
| rs6089904 | 20 | 62018289 | T | T | 0.047486 | -0.064 | 0.009 | 4.01E-12 | 48.10 |
| rs9607805 | 22 | 41854446 | T | T | 0.725 | 0.030 | 0.004 | 1.37E-11 | 45.70 |

SNP, single nucleotide polymorphism; A1, effect allele; A2, other allele; FRQ, effect allele frequency; SE, standard error;

**Supplementary Table 4: Characteristics of genetic instruments associated with smoking initiation.**

| SNP | CHR | BP | A1 | A2 | FRQ | beta | se | P | F |
| --- | --- | --- | --- | --- | --- | --- | --- | --- | --- |
| rs12130857 | 1 | 7791461 | A | G | 0.325 | -0.018 | 0.003 | 3.65E-11 | 43.80 |
| rs301807 | 1 | 8484823 | G | A | 0.57 | 0.018 | 0.003 | 2.5E-12 | 49.00 |
| rs3820277 | 1 | 18436657 | T | G | 0.526 | -0.019 | 0.003 | 1.57E-13 | 54.50 |
| rs1889571 | 1 | 32195819 | G | T | 0.131 | 0.022 | 0.004 | 4.19E-09 | 34.50 |
| rs10914684 | 1 | 33795572 | A | G | 0.324 | -0.016 | 0.003 | 6.32E-09 | 33.70 |
| rs2637869 | 1 | 38757237 | A | G | 0.297 | 0.018 | 0.003 | 6.54E-11 | 42.70 |
| rs12755632 | 1 | 41776623 | G | A | 0.316 | -0.015 | 0.003 | 1.93E-08 | 31.60 |
| rs951740 | 1 | 44011737 | A | G | 0.625 | 0.030 | 0.003 | 3.82E-29 | 126.00 |
| rs925524 | 1 | 46496709 | G | A | 0.71 | 0.016 | 0.003 | 2.94E-08 | 30.70 |
| rs12022778 | 1 | 50603995 | C | A | 0.202 | 0.027 | 0.003 | 3.18E-17 | 71.20 |
| rs11587399 | 1 | 50861071 | T | A | 0.221 | -0.018 | 0.003 | 7.25E-09 | 33.50 |
| rs4912332 | 1 | 58815243 | T | C | 0.491 | 0.014 | 0.003 | 2.94E-08 | 30.70 |
| rs1937443 | 1 | 66469643 | G | C | 0.563 | 0.020 | 0.003 | 1.79E-15 | 63.30 |
| rs1022528 | 1 | 71490122 | A | G | 0.344 | 0.017 | 0.003 | 8.48E-11 | 42.10 |
| rs12740789 | 1 | 72752073 | A | G | 0.178 | -0.028 | 0.003 | 1.18E-17 | 73.20 |
| rs80054503 | 1 | 72900406 | C | T | 0.116 | -0.024 | 0.004 | 3.1E-09 | 35.10 |
| rs10789369 | 1 | 73824909 | G | A | 0.615 | -0.023 | 0.003 | 3.39E-19 | 80.20 |
| rs1514176 | 1 | 74991596 | A | G | 0.58 | -0.019 | 0.003 | 7.67E-14 | 55.90 |
| rs10873871 | 1 | 76689019 | G | A | 0.207 | 0.017 | 0.003 | 2.82E-08 | 30.80 |
| rs11162019 | 1 | 87913176 | T | C | 0.363 | -0.015 | 0.003 | 5.06E-09 | 34.20 |
| rs1008078 | 1 | 91189731 | T | C | 0.402 | 0.023 | 0.003 | 1.63E-18 | 77.10 |
| rs1935571 | 1 | 96414335 | G | T | 0.48 | -0.016 | 0.003 | 6.99E-10 | 38.00 |
| rs12027999 | 1 | 154206358 | C | T | 0.12 | -0.024 | 0.004 | 5.33E-10 | 38.60 |
| rs45444697 | 1 | 155034632 | G | C | 0.212 | 0.020 | 0.003 | 2.72E-10 | 39.90 |
| rs2901785 | 1 | 174104743 | A | G | 0.446 | -0.017 | 0.003 | 1.47E-11 | 45.60 |
| rs147052174 | 1 | 179783167 | T | G | 0.0171 | 0.062 | 0.010 | 2.3E-10 | 40.20 |
| rs35656245 | 1 | 190957480 | A | G | 0.276 | 0.016 | 0.003 | 2.23E-08 | 31.30 |
| rs12739243 | 1 | 210302043 | C | T | 0.221 | -0.021 | 0.003 | 4.45E-12 | 47.90 |
| rs12563365 | 1 | 236872829 | A | G | 0.556 | 0.017 | 0.003 | 1.05E-10 | 41.70 |
| rs876793 | 1 | 237852083 | C | T | 0.3493 | -0.018 | 0.003 | 5.69E-11 | 42.90 |
| rs114976176 | 2 | 264621 | C | A | 0.3516 | -0.016 | 0.003 | 6.04E-09 | 33.80 |
| rs62106258 | 2 | 417167 | C | T | 0.0473 | -0.045 | 0.006 | 3.33E-14 | 57.50 |
| rs6731872 | 2 | 624205 | G | T | 0.826 | 0.032 | 0.003 | 5.35E-21 | 88.40 |
| rs1022376 | 2 | 22067213 | C | T | 0.5158 | -0.015 | 0.003 | 1.66E-08 | 31.90 |
| rs61533748 | 2 | 22582968 | C | T | 0.384 | 0.017 | 0.003 | 2.82E-11 | 44.30 |
| rs72790288 | 2 | 29513404 | A | G | 0.0282 | -0.046 | 0.008 | 3.28E-09 | 35.00 |
| rs2710634 | 2 | 32808804 | C | T | 0.521 | -0.018 | 0.003 | 3.36E-12 | 48.50 |
| rs62137126 | 2 | 44250149 | G | A | 0.1211 | -0.024 | 0.004 | 1.31E-09 | 36.80 |
| rs1004787 | 2 | 45159091 | A | G | 0.552 | 0.028 | 0.003 | 1.11E-28 | 123.00 |
| rs7598402 | 2 | 50735943 | G | C | 0.4921 | -0.015 | 0.003 | 7.38E-09 | 33.40 |
| rs10490159 | 2 | 51341259 | T | C | 0.394 | 0.017 | 0.003 | 3.86E-11 | 43.70 |
| rs1518393 | 2 | 58171220 | C | A | 0.619 | 0.017 | 0.003 | 1.3E-10 | 41.30 |
| rs17616642 | 2 | 59022210 | G | A | 0.2469 | -0.017 | 0.003 | 2.1E-08 | 31.40 |
| rs6730325 | 2 | 59315828 | A | G | 0.6098 | -0.015 | 0.003 | 2.1E-08 | 31.40 |
| rs2539706 | 2 | 59819545 | A | G | 0.5299 | 0.016 | 0.003 | 1.95E-10 | 40.50 |
| rs7585579 | 2 | 60024857 | G | C | 0.499 | 0.020 | 0.003 | 5.48E-15 | 61.10 |
| rs1863161 | 2 | 60139524 | A | G | 0.5609 | 0.015 | 0.003 | 2.34E-09 | 35.70 |
| rs359247 | 2 | 60477052 | T | A | 0.6387 | 0.022 | 0.003 | 9.89E-17 | 69.00 |
| rs62180324 | 2 | 63416606 | A | G | 0.212 | -0.020 | 0.003 | 3.91E-10 | 39.20 |
| rs6750107 | 2 | 80748807 | A | G | 0.3869 | 0.015 | 0.003 | 2.6E-08 | 31.00 |
| rs12714017 | 2 | 80999398 | C | T | 0.511 | 0.015 | 0.003 | 3.65E-09 | 34.80 |
| rs56208390 | 2 | 83247997 | G | A | 0.123 | 0.022 | 0.004 | 2.68E-08 | 30.90 |
| rs11692435 | 2 | 98275354 | A | G | 0.0848 | 0.025 | 0.005 | 4.47E-08 | 29.90 |
| rs13392222 | 2 | 100672408 | C | A | 0.139 | -0.023 | 0.004 | 1.93E-10 | 40.50 |
| rs1901477 | 2 | 104126983 | G | A | 0.511 | 0.030 | 0.003 | 2.07E-31 | 136.00 |
| rs11889814 | 2 | 104432494 | C | A | 0.128 | -0.021 | 0.004 | 3.44E-08 | 30.40 |
| rs3811038 | 2 | 113240183 | C | T | 0.279 | 0.019 | 0.003 | 1.58E-11 | 45.40 |
| rs75210106 | 2 | 113246436 | T | C | 0.1767 | -0.019 | 0.003 | 2.33E-08 | 31.20 |
| rs34399632 | 2 | 137571174 | G | A | 0.232 | 0.019 | 0.003 | 1.46E-10 | 41.10 |
| rs74697736 | 2 | 145412271 | A | G | 0.2872 | 0.022 | 0.003 | 2.43E-15 | 62.70 |
| rs6756212 | 2 | 146140132 | T | C | 0.535 | -0.034 | 0.003 | 3.49E-40 | 176.00 |
| rs3076896 | 2 | 146283610 | A | G | 0.3899 | 0.023 | 0.003 | 1.99E-16 | 67.60 |
| rs16826827 | 2 | 147825689 | C | T | 0.124 | -0.022 | 0.004 | 9.17E-09 | 33.00 |
| rs1445649 | 2 | 155682556 | C | T | 0.538 | 0.021 | 0.003 | 8.48E-16 | 64.80 |
| rs1722666 | 2 | 161816880 | T | C | 0.732 | 0.016 | 0.003 | 2.17E-08 | 31.30 |
| rs11678980 | 2 | 162101261 | A | G | 0.45 | 0.018 | 0.003 | 5.19E-12 | 47.60 |
| rs12474587 | 2 | 162802993 | T | G | 0.429 | 0.024 | 0.003 | 4.83E-21 | 88.60 |
| rs357304 | 2 | 164862639 | C | T | 0.727 | 0.017 | 0.003 | 5.4E-09 | 34.00 |
| rs13007361 | 2 | 166250244 | A | G | 0.208 | 0.018 | 0.003 | 2.29E-08 | 31.20 |
| rs7600835 | 2 | 172521827 | A | G | 0.342 | -0.015 | 0.003 | 1.8E-08 | 31.70 |
| rs6750529 | 2 | 182027603 | T | C | 0.744 | 0.020 | 0.003 | 9.26E-12 | 46.50 |
| rs17229285 | 2 | 199523122 | T | C | 0.505 | -0.015 | 0.003 | 1.27E-09 | 36.90 |
| rs3115418 | 2 | 200936399 | C | T | 0.454 | -0.014 | 0.003 | 2.79E-08 | 30.90 |
| rs62193862 | 2 | 202843875 | A | G | 0.0999 | 0.024 | 0.004 | 1.99E-08 | 31.50 |
| rs4674916 | 2 | 225365635 | A | C | 0.3277 | -0.018 | 0.003 | 3.06E-11 | 44.10 |
| rs4674993 | 2 | 226332033 | G | A | 0.2 | -0.024 | 0.003 | 4.85E-14 | 56.80 |
| rs11713899 | 3 | 2365026 | C | A | 0.171 | 0.019 | 0.003 | 3.15E-08 | 30.60 |
| rs748832 | 3 | 16851202 | G | A | 0.371 | 0.017 | 0.003 | 6.6E-11 | 42.60 |
| rs10446419 | 3 | 25725501 | G | A | 0.207 | -0.020 | 0.003 | 5.05E-10 | 38.70 |
| rs13319205 | 3 | 47800216 | A | T | 0.29 | 0.017 | 0.003 | 3.77E-09 | 34.70 |
| rs3172494 | 3 | 48731487 | T | G | 0.115 | -0.029 | 0.004 | 3.4E-13 | 53.00 |
| rs2526390 | 3 | 50192760 | T | C | 0.334 | 0.020 | 0.003 | 3.62E-14 | 57.40 |
| rs2276825 | 3 | 52886605 | C | T | 0.245 | 0.019 | 0.003 | 1.89E-10 | 40.60 |
| rs2306866 | 3 | 53766212 | T | A | 0.614 | -0.017 | 0.003 | 1.89E-10 | 40.60 |
| rs73831818 | 3 | 55988394 | G | A | 0.057 | 0.032 | 0.005 | 5.46E-09 | 34.00 |
| rs1910236 | 3 | 59434420 | A | G | 0.469 | 0.015 | 0.003 | 9.91E-09 | 32.90 |
| rs7640107 | 3 | 59966156 | T | C | 0.4308 | -0.014 | 0.003 | 3.46E-08 | 30.40 |
| rs2734390 | 3 | 60459291 | G | A | 0.372 | 0.015 | 0.003 | 2.09E-08 | 31.40 |
| rs221988 | 3 | 64234307 | C | A | 0.384 | -0.015 | 0.003 | 1.43E-08 | 32.20 |
| rs2196356 | 3 | 70890288 | C | G | 0.2889 | -0.019 | 0.003 | 2.45E-11 | 44.60 |
| rs11128203 | 3 | 71064431 | A | T | 0.53 | 0.020 | 0.003 | 1.29E-15 | 63.90 |
| rs62246017 | 3 | 71483084 | A | G | 0.3226 | -0.016 | 0.003 | 3.03E-09 | 35.20 |
| rs4543050 | 3 | 74954560 | T | A | 0.816 | 0.022 | 0.003 | 1.45E-11 | 45.60 |
| rs6782116 | 3 | 77176032 | T | C | 0.415 | -0.015 | 0.003 | 1.46E-08 | 32.10 |
| rs13066050 | 3 | 81325861 | T | C | 0.208 | 0.019 | 0.003 | 1.93E-09 | 36.00 |
| rs12633090 | 3 | 83241365 | C | G | 0.182 | -0.023 | 0.003 | 3.16E-12 | 48.60 |
| rs1549979 | 3 | 85460131 | T | C | 0.615 | -0.025 | 0.003 | 8.8E-21 | 87.40 |
| rs74664784 | 3 | 85475292 | C | T | 0.376 | -0.020 | 0.003 | 9.34E-13 | 51.00 |
| rs57153235 | 3 | 85902536 | G | T | 0.318 | -0.019 | 0.003 | 1.56E-12 | 50.00 |
| rs6437769 | 3 | 107997514 | T | C | 0.581 | 0.014 | 0.003 | 3.74E-08 | 30.30 |
| rs9288999 | 3 | 114147927 | A | G | 0.735 | 0.017 | 0.003 | 1.5E-09 | 36.50 |
| rs6438436 | 3 | 117822149 | T | C | 0.816 | 0.025 | 0.003 | 5.33E-14 | 56.60 |
| rs12053870 | 3 | 118302515 | G | T | 0.5415 | 0.016 | 0.003 | 1.02E-09 | 37.30 |
| rs9826984 | 3 | 131945722 | A | G | 0.542 | -0.014 | 0.003 | 3.87E-08 | 30.20 |
| rs2279829 | 3 | 147106319 | T | C | 0.216 | -0.017 | 0.003 | 2.05E-08 | 31.50 |
| rs2319545 | 3 | 147719648 | A | C | 0.1491 | 0.023 | 0.004 | 8.3E-11 | 42.20 |
| rs10935779 | 3 | 149543102 | T | C | 0.415 | -0.014 | 0.003 | 2.95E-08 | 30.70 |
| rs963354 | 3 | 157393770 | A | C | 0.687 | 0.015 | 0.003 | 4.21E-08 | 30.00 |
| rs1714521 | 3 | 158284861 | C | A | 0.411 | -0.016 | 0.003 | 3.07E-10 | 39.60 |
| rs1449012 | 3 | 159048333 | T | C | 0.463 | -0.015 | 0.003 | 1.77E-09 | 36.20 |
| rs9850597 | 3 | 161761866 | A | G | 0.816 | -0.019 | 0.003 | 1.65E-08 | 31.90 |
| rs1187820 | 3 | 173072584 | T | C | 0.439 | -0.014 | 0.003 | 2.69E-08 | 30.90 |
| rs16828799 | 3 | 173353739 | T | G | 0.156 | 0.020 | 0.004 | 1.83E-08 | 31.70 |
| rs9841807 | 3 | 175718927 | T | C | 0.273 | 0.016 | 0.003 | 1.35E-08 | 32.30 |
| rs7631379 | 3 | 181409057 | C | T | 0.206 | 0.021 | 0.003 | 3.94E-11 | 43.60 |
| rs4140932 | 4 | 15458598 | A | T | 0.431 | -0.014 | 0.003 | 4.89E-08 | 29.80 |
| rs12642744 | 4 | 28027176 | T | G | 0.744 | -0.017 | 0.003 | 2.82E-08 | 30.80 |
| rs59537158 | 4 | 28246049 | T | C | 0.214 | 0.022 | 0.003 | 4.62E-13 | 52.40 |
| rs1389171 | 4 | 28822284 | A | T | 0.241 | -0.017 | 0.003 | 4.45E-09 | 34.40 |
| rs55944129 | 4 | 29082156 | C | T | 0.267 | -0.018 | 0.003 | 1.06E-09 | 37.20 |
| rs58400863 | 4 | 31184484 | A | G | 0.347 | -0.020 | 0.003 | 4.89E-14 | 56.80 |
| rs7657022 | 4 | 35501032 | G | A | 0.489 | 0.018 | 0.003 | 7.34E-13 | 51.50 |
| rs55900829 | 4 | 35514712 | T | A | 0.3348 | 0.019 | 0.003 | 5.63E-12 | 47.50 |
| rs112725451 | 4 | 68017710 | T | C | 0.169 | 0.026 | 0.003 | 1.65E-14 | 58.90 |
| rs1160685 | 4 | 94052854 | G | C | 0.45 | 0.015 | 0.003 | 2.31E-09 | 35.70 |
| rs1435479 | 4 | 94550450 | T | G | 0.2875 | 0.016 | 0.003 | 5.68E-09 | 33.90 |
| rs3934797 | 4 | 112467612 | A | G | 0.182 | -0.021 | 0.003 | 1.12E-10 | 41.60 |
| rs71602617 | 4 | 136406155 | T | C | 0.216 | -0.018 | 0.003 | 2.1E-08 | 31.40 |
| rs7696257 | 4 | 137474783 | A | G | 0.366 | 0.015 | 0.003 | 6.78E-09 | 33.60 |
| rs13109980 | 4 | 140886963 | A | G | 0.326 | -0.022 | 0.003 | 3.37E-16 | 66.60 |
| rs1116690 | 4 | 143510148 | G | A | 0.742 | 0.016 | 0.003 | 2.16E-08 | 31.30 |
| rs13110073 | 4 | 147797913 | C | T | 0.395 | -0.025 | 0.003 | 3.24E-21 | 89.40 |
| rs28717373 | 4 | 147985231 | T | C | 0.3562 | -0.016 | 0.003 | 6.16E-10 | 38.30 |
| rs62340589 | 4 | 176875795 | C | G | 0.201 | 0.017 | 0.003 | 4.31E-08 | 30.00 |
| rs12517438 | 5 | 30842054 | G | T | 0.538 | 0.015 | 0.003 | 1.89E-09 | 36.10 |
| rs35375873 | 5 | 43190647 | C | G | 0.11 | -0.027 | 0.004 | 3.29E-11 | 44.00 |
| rs986714 | 5 | 50821338 | T | A | 0.445 | -0.016 | 0.003 | 4.13E-10 | 39.10 |
| rs71592686 | 5 | 60121271 | C | T | 0.274 | 0.021 | 0.003 | 3.85E-13 | 52.70 |
| rs2028269 | 5 | 79308315 | A | G | 0.399 | 0.016 | 0.003 | 5.19E-10 | 38.60 |
| rs6874731 | 5 | 80263865 | G | T | 0.484 | 0.015 | 0.003 | 1.83E-09 | 36.10 |
| rs6452785 | 5 | 87685500 | T | C | 0.474 | -0.027 | 0.003 | 4.69E-26 | 111.00 |
| rs10805858 | 5 | 88873832 | T | A | 0.3353 | 0.018 | 0.003 | 1.88E-11 | 45.10 |
| rs181508347 | 5 | 91366274 | G | T | 0.0097 | 0.081 | 0.013 | 4.95E-10 | 38.70 |
| rs42417 | 5 | 94198290 | T | C | 0.691 | 0.017 | 0.003 | 8.27E-10 | 37.70 |
| rs72780746 | 5 | 103929588 | C | T | 0.173 | -0.026 | 0.003 | 2.05E-14 | 58.50 |
| rs10060196 | 5 | 106455988 | A | C | 0.5806 | 0.018 | 0.003 | 1.29E-12 | 50.30 |
| rs72789626 | 5 | 106825618 | A | T | 0.136 | -0.026 | 0.004 | 5.13E-12 | 47.60 |
| rs17165769 | 5 | 107365642 | G | A | 0.3949 | 0.016 | 0.003 | 9.56E-10 | 37.40 |
| rs329124 | 5 | 133865452 | G | A | 0.428 | -0.016 | 0.003 | 1.96E-10 | 40.50 |
| rs1385108 | 5 | 154839646 | T | C | 0.239 | 0.019 | 0.003 | 3.84E-10 | 39.20 |
| rs1173461 | 5 | 157707571 | T | C | 0.327 | 0.017 | 0.003 | 9.51E-10 | 37.40 |
| rs11956866 | 5 | 161018271 | G | T | 0.567 | -0.015 | 0.003 | 7.82E-09 | 33.30 |
| rs3909281 | 5 | 165096435 | G | T | 0.536 | 0.021 | 0.003 | 1.62E-16 | 68.00 |
| rs3843905 | 5 | 165427280 | T | C | 0.403 | -0.015 | 0.003 | 5.41E-09 | 34.00 |
| rs79476395 | 5 | 166063680 | G | A | 0.0726 | 0.033 | 0.005 | 1.04E-11 | 46.20 |
| rs6890961 | 5 | 166778503 | T | C | 0.624 | -0.019 | 0.003 | 2.13E-13 | 53.90 |
| rs4044321 | 5 | 166989513 | G | A | 0.644 | -0.023 | 0.003 | 1.75E-17 | 72.40 |
| rs2173019 | 5 | 167614971 | A | T | 0.177 | 0.028 | 0.003 | 2.98E-17 | 71.40 |
| rs10042827 | 5 | 170299916 | C | T | 0.681 | 0.017 | 0.003 | 9.41E-10 | 37.40 |
| rs359431 | 5 | 173288534 | T | C | 0.56 | -0.014 | 0.003 | 3.16E-08 | 30.60 |
| rs1059490 | 6 | 26171250 | C | T | 0.367 | -0.019 | 0.003 | 2.16E-12 | 49.30 |
| rs6932350 | 6 | 26571629 | A | T | 0.4547 | 0.015 | 0.003 | 5.13E-09 | 34.10 |
| rs1150668 | 6 | 28129789 | G | T | 0.419 | -0.019 | 0.003 | 8.54E-13 | 51.20 |
| rs1632941 | 6 | 29796685 | C | T | 0.46 | -0.016 | 0.003 | 6.67E-10 | 38.10 |
| rs3218116 | 6 | 41901763 | T | C | 0.256 | -0.020 | 0.003 | 1.05E-11 | 46.20 |
| rs160631 | 6 | 52895230 | G | T | 0.731 | -0.017 | 0.003 | 1.87E-09 | 36.10 |
| rs7743165 | 6 | 67521222 | G | T | 0.495 | 0.019 | 0.003 | 4.15E-14 | 57.10 |
| rs79180767 | 6 | 67540984 | T | C | 0.253 | 0.020 | 0.003 | 7E-12 | 47.00 |
| rs10945141 | 6 | 69470709 | A | G | 0.263 | 0.018 | 0.003 | 3.59E-10 | 39.30 |
| rs17554906 | 6 | 92226609 | C | G | 0.444 | 0.014 | 0.003 | 3.14E-08 | 30.60 |
| rs619087 | 6 | 94175279 | G | A | 0.422 | 0.014 | 0.003 | 3.1E-08 | 30.60 |
| rs6568832 | 6 | 97702876 | A | G | 0.7539 | 0.019 | 0.003 | 1.74E-10 | 40.70 |
| rs12195240 | 6 | 98636905 | A | G | 0.285 | 0.025 | 0.003 | 1.08E-18 | 77.90 |
| rs6936160 | 6 | 100347745 | T | C | 0.698 | 0.020 | 0.003 | 4.2E-13 | 52.50 |
| rs12530388 | 6 | 101329173 | C | A | 0.511 | -0.018 | 0.003 | 5.83E-13 | 51.90 |
| rs3800227 | 6 | 108994161 | G | A | 0.742 | 0.017 | 0.003 | 3.64E-09 | 34.80 |
| rs118202 | 6 | 111658371 | T | G | 0.812 | -0.037 | 0.003 | 1.9E-29 | 127.00 |
| rs73008357 | 6 | 156431856 | C | A | 0.121 | -0.022 | 0.004 | 2.44E-08 | 31.10 |
| rs9331343 | 6 | 157738258 | C | T | 0.568 | -0.014 | 0.003 | 3.9E-08 | 30.20 |
| rs10698713 | 6 | 158882320 | A | G | 0.0544 | -0.034 | 0.006 | 2.38E-09 | 35.60 |
| rs1737329 | 6 | 163807748 | G | C | 0.742 | 0.017 | 0.003 | 5.08E-09 | 34.20 |
| rs10272990 | 7 | 1703675 | C | T | 0.3276 | -0.021 | 0.003 | 1.27E-14 | 59.40 |
| rs6948707 | 7 | 1870794 | G | T | 0.419 | 0.024 | 0.003 | 4.24E-21 | 88.90 |
| rs10259715 | 7 | 3329967 | A | T | 0.2099 | -0.019 | 0.003 | 6.42E-09 | 33.70 |
| rs13237637 | 7 | 3503207 | C | G | 0.485 | -0.024 | 0.003 | 1.54E-20 | 86.30 |
| rs79631993 | 7 | 69432311 | C | A | 0.2163 | -0.017 | 0.003 | 3.67E-08 | 30.30 |
| rs7809303 | 7 | 69484366 | A | G | 0.325 | -0.021 | 0.003 | 3.48E-15 | 62.00 |
| rs7802996 | 7 | 77771983 | T | C | 0.166 | -0.021 | 0.003 | 1.06E-09 | 37.20 |
| rs1030015 | 7 | 78139581 | T | G | 0.5196 | 0.014 | 0.003 | 2.15E-08 | 31.40 |
| rs4727189 | 7 | 88442568 | C | T | 0.344 | 0.015 | 0.003 | 0.00000003 | 30.70 |
| rs76841737 | 7 | 91281409 | G | C | 0.103 | -0.023 | 0.004 | 3.26E-08 | 30.50 |
| rs11768481 | 7 | 96629103 | A | C | 0.34 | -0.019 | 0.003 | 5.23E-12 | 47.60 |
| rs1799068 | 7 | 97707069 | T | G | 0.379 | 0.017 | 0.003 | 2.59E-10 | 40.00 |
| rs13437771 | 7 | 99071478 | G | A | 0.155 | -0.027 | 0.004 | 1.39E-14 | 59.30 |
| rs11766326 | 7 | 111100585 | C | T | 0.506 | -0.018 | 0.003 | 1.79E-11 | 45.20 |
| rs6968380 | 7 | 114940159 | A | G | 0.681 | -0.023 | 0.003 | 1.05E-17 | 73.40 |
| rs112913817 | 7 | 115077394 | G | A | 0.0113 | 0.078 | 0.012 | 9.28E-11 | 42.00 |
| rs10233018 | 7 | 117523709 | G | A | 0.516 | 0.025 | 0.003 | 4.77E-22 | 93.20 |
| rs10953957 | 7 | 121954709 | A | G | 0.386 | 0.014 | 0.003 | 3.66E-08 | 30.30 |
| rs77283305 | 7 | 132593831 | A | G | 0.3058 | -0.015 | 0.003 | 3.91E-08 | 30.20 |
| rs10279261 | 7 | 133589846 | A | G | 0.618 | -0.019 | 0.003 | 6.05E-13 | 51.80 |
| rs1561112 | 7 | 133840652 | C | T | 0.4128 | -0.015 | 0.003 | 3.84E-09 | 34.70 |
| rs2952251 | 8 | 10143164 | G | A | 0.7444 | 0.016 | 0.003 | 4.24E-08 | 30.00 |
| rs4326350 | 8 | 10763655 | G | C | 0.493 | -0.018 | 0.003 | 5.16E-12 | 47.60 |
| rs11780471 | 8 | 27344719 | A | G | 0.0631 | -0.039 | 0.005 | 1.57E-13 | 54.50 |
| rs11783093 | 8 | 27425349 | T | C | 0.158 | -0.047 | 0.003 | 2.07E-41 | 182.00 |
| rs1565735 | 8 | 27426077 | A | T | 0.2045 | -0.019 | 0.003 | 1.33E-09 | 36.80 |
| rs7836565 | 8 | 52569449 | T | C | 0.718 | -0.016 | 0.003 | 4.36E-08 | 30.00 |
| rs13261666 | 8 | 59814666 | T | G | 0.517 | -0.020 | 0.003 | 4.36E-15 | 61.50 |
| rs3850736 | 8 | 64912021 | G | C | 0.474 | 0.019 | 0.003 | 6.43E-14 | 56.20 |
| rs2063976 | 8 | 91096366 | T | C | 0.665 | -0.020 | 0.003 | 7.45E-14 | 55.90 |
| rs6993429 | 8 | 92733282 | A | C | 0.453 | -0.019 | 0.003 | 9.87E-14 | 55.40 |
| rs6986430 | 8 | 93048104 | C | T | 0.2224 | -0.024 | 0.003 | 1.99E-15 | 63.10 |
| rs9987376 | 8 | 93190014 | G | T | 0.5743 | -0.020 | 0.003 | 2.01E-15 | 63.10 |
| rs290601 | 8 | 115374642 | T | C | 0.274 | 0.016 | 0.003 | 1.14E-08 | 32.60 |
| rs3847244 | 9 | 3025368 | T | C | 0.47 | 0.019 | 0.003 | 2.6E-13 | 53.50 |
| rs11791671 | 9 | 3398679 | T | C | 0.0673 | 0.028 | 0.005 | 4.24E-08 | 30.00 |
| rs7024924 | 9 | 8282399 | C | T | 0.174 | 0.019 | 0.003 | 1.9E-08 | 31.60 |
| rs6474609 | 9 | 10981069 | A | T | 0.5867 | -0.016 | 0.003 | 1.71E-09 | 36.30 |
| rs1931431 | 9 | 11161799 | C | G | 0.478 | 0.018 | 0.003 | 8.56E-13 | 51.10 |
| rs7867822 | 9 | 20676454 | G | A | 0.673 | -0.015 | 0.003 | 2.76E-08 | 30.90 |
| rs10966092 | 9 | 23831658 | C | T | 0.267 | -0.020 | 0.003 | 1.12E-12 | 50.60 |
| rs10969352 | 9 | 29747488 | A | T | 0.5 | 0.014 | 0.003 | 1.82E-08 | 31.70 |
| rs4877285 | 9 | 81354129 | A | G | 0.6682 | -0.018 | 0.003 | 2.1E-11 | 44.90 |
| rs1930371 | 9 | 81444104 | T | C | 0.241 | -0.017 | 0.003 | 7.09E-09 | 33.50 |
| rs2378662 | 9 | 86707289 | A | G | 0.541 | 0.015 | 0.003 | 2.67E-09 | 35.40 |
| rs1927901 | 9 | 120519111 | C | T | 0.553 | -0.014 | 0.003 | 3.1E-08 | 30.60 |
| rs4837631 | 9 | 122061948 | T | C | 0.446 | -0.015 | 0.003 | 2.03E-09 | 35.90 |
| rs1759433 | 9 | 128073097 | A | G | 0.48 | 0.015 | 0.003 | 1.69E-09 | 36.30 |
| rs34553878 | 9 | 134334588 | G | A | 0.111 | 0.025 | 0.004 | 1.17E-09 | 37.00 |
| rs7026534 | 9 | 134907263 | G | T | 0.7038 | -0.017 | 0.003 | 2.68E-09 | 35.40 |
| rs10858334 | 9 | 137989785 | G | C | 0.14 | 0.023 | 0.004 | 1.18E-09 | 37.00 |
| rs10905461 | 10 | 8803551 | C | T | 0.748 | -0.016 | 0.003 | 2.36E-08 | 31.20 |
| rs7920501 | 10 | 10043159 | A | T | 0.465 | -0.016 | 0.003 | 1.25E-09 | 36.90 |
| rs1291821 | 10 | 11133823 | G | A | 0.534 | 0.014 | 0.003 | 1.39E-08 | 32.20 |
| rs11258417 | 10 | 13533053 | T | C | 0.391 | -0.015 | 0.003 | 2.71E-08 | 30.90 |
| rs7072776 | 10 | 22032942 | G | A | 0.712 | -0.022 | 0.003 | 5.66E-15 | 61.00 |
| rs2796793 | 10 | 36634124 | A | G | 0.452 | 0.014 | 0.003 | 1.55E-08 | 32.00 |
| rs1733760 | 10 | 56698174 | C | T | 0.51 | 0.015 | 0.003 | 6.7E-09 | 33.60 |
| rs7921378 | 10 | 63674885 | C | G | 0.482 | -0.023 | 0.003 | 6.1E-20 | 83.60 |
| rs7901883 | 10 | 103186838 | A | G | 0.2303 | -0.019 | 0.003 | 1.98E-10 | 40.50 |
| rs11594623 | 10 | 103960351 | C | T | 0.2342 | 0.027 | 0.003 | 7.45E-20 | 83.20 |
| rs11191269 | 10 | 104120522 | G | C | 0.1933 | 0.018 | 0.003 | 4.61E-08 | 29.90 |
| rs28408682 | 10 | 104403310 | G | A | 0.6 | 0.017 | 0.003 | 1.41E-10 | 41.10 |
| rs12244388 | 10 | 104640052 | A | G | 0.35 | 0.026 | 0.003 | 4.31E-22 | 93.40 |
| rs111842178 | 10 | 104852121 | G | A | 0.231 | 0.022 | 0.003 | 2.24E-12 | 49.30 |
| rs34970111 | 10 | 106078937 | T | C | 0.458 | -0.015 | 0.003 | 1.28E-08 | 32.40 |
| rs9787523 | 10 | 106460460 | C | T | 0.418 | -0.016 | 0.003 | 1.42E-09 | 36.60 |
| rs11192347 | 10 | 106929313 | A | G | 0.104 | -0.026 | 0.004 | 6.15E-10 | 38.30 |
| rs10885480 | 10 | 115378364 | C | T | 0.284 | -0.019 | 0.003 | 3.83E-11 | 43.70 |
| rs4752018 | 10 | 118678712 | A | C | 0.231 | 0.019 | 0.003 | 4.42E-10 | 38.90 |
| rs9423279 | 10 | 125680419 | G | C | 0.645 | -0.019 | 0.003 | 3.06E-12 | 48.70 |
| rs6265 | 11 | 27679916 | T | C | 0.188 | -0.029 | 0.003 | 2.81E-19 | 80.60 |
| rs4275621 | 11 | 28652996 | G | A | 0.382 | -0.021 | 0.003 | 3.76E-16 | 66.40 |
| rs62618693 | 11 | 32956492 | T | C | 0.0428 | -0.035 | 0.006 | 2.09E-08 | 31.40 |
| rs2939756 | 11 | 41436297 | A | G | 0.48 | -0.016 | 0.003 | 7.45E-10 | 37.90 |
| rs1381775 | 11 | 42442826 | C | T | 0.712 | -0.016 | 0.003 | 2.79E-08 | 30.80 |
| rs2959084 | 11 | 46078656 | A | G | 0.7047 | 0.017 | 0.003 | 9.82E-10 | 37.40 |
| rs3740977 | 11 | 46393574 | C | T | 0.167 | 0.019 | 0.003 | 1.17E-08 | 32.50 |
| rs61886926 | 11 | 64133552 | T | C | 0.384 | -0.018 | 0.003 | 7.3E-12 | 46.90 |
| rs61884449 | 11 | 64485193 | T | C | 0.1492 | 0.020 | 0.004 | 2.32E-08 | 31.20 |
| rs644740 | 11 | 65561468 | T | C | 0.457 | -0.014 | 0.003 | 3.67E-08 | 30.30 |
| rs7943721 | 11 | 73309393 | A | G | 0.829 | -0.021 | 0.003 | 3.58E-10 | 39.30 |
| rs7929518 | 11 | 85980958 | G | A | 0.773 | 0.019 | 0.003 | 2.55E-10 | 40.00 |
| rs586699 | 11 | 92289734 | A | G | 0.543 | -0.015 | 0.003 | 7.29E-09 | 33.50 |
| rs76460663 | 11 | 111979741 | G | C | 0.0411 | -0.042 | 0.006 | 4.15E-11 | 43.50 |
| rs2155646 | 11 | 112912811 | C | T | 0.4 | 0.038 | 0.003 | 9.44E-48 | 211.00 |
| rs78239456 | 11 | 112984491 | T | A | 0.3765 | -0.018 | 0.003 | 9.37E-12 | 46.50 |
| rs1713676 | 11 | 113660576 | G | A | 0.5225 | -0.017 | 0.003 | 5.38E-11 | 43.00 |
| rs238896 | 11 | 113994505 | A | G | 0.49 | -0.017 | 0.003 | 3.65E-11 | 43.80 |
| rs540860 | 11 | 121530888 | G | A | 0.543 | 0.018 | 0.003 | 5.75E-12 | 47.40 |
| rs1944689 | 11 | 121634334 | T | G | 0.7859 | 0.018 | 0.003 | 1.27E-08 | 32.40 |
| rs1834306 | 11 | 122023187 | G | A | 0.5794 | -0.014 | 0.003 | 1.96E-08 | 31.50 |
| rs1106363 | 11 | 131966264 | T | C | 0.3446 | 0.017 | 0.003 | 9.2E-11 | 42.00 |
| rs2010921 | 11 | 132098205 | A | G | 0.311 | 0.017 | 0.003 | 2.47E-10 | 40.10 |
| rs11057005 | 12 | 16748721 | G | A | 0.441 | -0.016 | 0.003 | 9.12E-10 | 37.50 |
| rs13906 | 12 | 49952394 | T | C | 0.109 | -0.025 | 0.004 | 1.98E-09 | 36.00 |
| rs4759229 | 12 | 56474480 | G | A | 0.656 | 0.016 | 0.003 | 6.53E-09 | 33.70 |
| rs7969559 | 12 | 69655167 | G | A | 0.713 | -0.017 | 0.003 | 1.53E-09 | 36.50 |
| rs7134009 | 12 | 75263193 | C | T | 0.287 | -0.016 | 0.003 | 4.3E-08 | 30.00 |
| rs77215829 | 12 | 112618346 | C | A | 0.131 | -0.024 | 0.004 | 2.02E-10 | 40.40 |
| rs1109480 | 12 | 121083279 | A | G | 0.384 | -0.017 | 0.003 | 1.84E-10 | 40.60 |
| rs11611651 | 12 | 133380790 | A | G | 0.0868 | 0.027 | 0.005 | 2.05E-09 | 35.90 |
| rs17197663 | 13 | 38172867 | A | G | 0.125 | -0.022 | 0.004 | 2.06E-08 | 31.40 |
| rs4264267 | 13 | 38359676 | T | C | 0.527 | 0.015 | 0.003 | 6.82E-09 | 33.60 |
| rs61959481 | 13 | 55834929 | A | G | 0.21 | -0.020 | 0.003 | 7.95E-11 | 42.30 |
| rs3098272 | 13 | 55931424 | C | A | 0.7988 | -0.018 | 0.003 | 2.08E-08 | 31.40 |
| rs9538162 | 13 | 59265043 | C | T | 0.4159 | 0.017 | 0.003 | 1.76E-11 | 45.20 |
| rs1413119 | 13 | 59339281 | T | C | 0.3963 | -0.015 | 0.003 | 4.77E-09 | 34.30 |
| rs56367474 | 13 | 59454139 | T | C | 0.304 | -0.017 | 0.003 | 4.2E-10 | 39.00 |
| rs55786907 | 13 | 59871584 | G | A | 0.1625 | 0.019 | 0.003 | 1.84E-08 | 31.70 |
| rs4886207 | 13 | 60705792 | C | T | 0.637 | -0.016 | 0.003 | 8.78E-10 | 37.60 |
| rs9540731 | 13 | 66949370 | T | C | 0.509 | -0.018 | 0.003 | 3.42E-12 | 48.40 |
| rs9545155 | 13 | 80191873 | C | T | 0.478 | -0.016 | 0.003 | 3.04E-10 | 39.70 |
| rs1772572 | 13 | 81191176 | A | C | 0.3241 | -0.017 | 0.003 | 5.62E-10 | 38.40 |
| rs75674569 | 13 | 96823724 | A | G | 0.0997 | -0.025 | 0.004 | 2.58E-09 | 35.50 |
| rs7333559 | 13 | 100546450 | A | G | 0.783 | -0.023 | 0.003 | 5.94E-14 | 56.40 |
| rs1108130 | 13 | 100648356 | A | T | 0.212 | 0.024 | 0.003 | 1.57E-14 | 59.00 |
| rs12855717 | 13 | 101252635 | T | C | 0.538 | 0.016 | 0.003 | 1.22E-09 | 36.90 |
| rs12878369 | 14 | 28346502 | A | C | 0.4148 | 0.017 | 0.003 | 1.6E-11 | 45.40 |
| rs2145451 | 14 | 29316842 | C | T | 0.193 | -0.020 | 0.003 | 5.44E-10 | 38.50 |
| rs9323328 | 14 | 58653514 | G | A | 0.537 | -0.014 | 0.003 | 2.55E-08 | 31.00 |
| rs1811739 | 14 | 77529375 | A | G | 0.248 | 0.018 | 0.003 | 5.97E-10 | 38.30 |
| rs8005334 | 14 | 79563654 | G | T | 0.36 | 0.017 | 0.003 | 3.44E-10 | 39.40 |
| rs34940743 | 14 | 80102233 | G | A | 0.346 | 0.016 | 0.003 | 2.8E-09 | 35.30 |
| rs2925128 | 14 | 98362355 | T | C | 0.3852 | 0.017 | 0.003 | 3.67E-10 | 39.30 |
| rs1381287 | 14 | 98597552 | T | C | 0.467 | 0.018 | 0.003 | 1.81E-12 | 49.70 |
| rs55913542 | 14 | 99693843 | T | G | 0.175 | 0.019 | 0.003 | 3.25E-08 | 30.60 |
| rs1435672 | 15 | 36399479 | C | T | 0.56 | 0.014 | 0.003 | 3.82E-08 | 30.20 |
| rs281296 | 15 | 47685010 | A | G | 0.357 | 0.025 | 0.003 | 1.59E-20 | 86.20 |
| rs1435741 | 15 | 47935843 | A | G | 0.433 | 0.018 | 0.003 | 1.09E-12 | 50.70 |
| rs56902655 | 15 | 63898709 | G | T | 0.136 | -0.022 | 0.004 | 4.09E-09 | 34.60 |
| rs2289791 | 15 | 67476952 | T | G | 0.247 | -0.018 | 0.003 | 2.01E-09 | 36.00 |
| rs60833441 | 15 | 74048768 | G | A | 0.461 | -0.014 | 0.003 | 2.28E-08 | 31.20 |
| rs62007780 | 15 | 78025464 | T | G | 0.416 | -0.016 | 0.003 | 7.48E-10 | 37.90 |
| rs12442563 | 15 | 83893243 | T | G | 0.223 | -0.023 | 0.003 | 3.13E-14 | 57.60 |
| rs4310804 | 15 | 96858409 | G | C | 0.247 | -0.018 | 0.003 | 7.55E-10 | 37.90 |
| rs8027457 | 15 | 99204101 | C | T | 0.511 | 0.015 | 0.003 | 1.88E-09 | 36.10 |
| rs1139897 | 16 | 720986 | A | G | 0.23 | -0.024 | 0.003 | 1.77E-15 | 63.30 |
| rs11076962 | 16 | 5811367 | C | T | 0.279 | 0.018 | 0.003 | 1.2E-10 | 41.50 |
| rs7192140 | 16 | 10173748 | C | T | 0.498 | -0.017 | 0.003 | 3.4E-11 | 43.90 |
| rs9922607 | 16 | 17570220 | T | C | 0.2 | -0.022 | 0.003 | 3.42E-12 | 48.40 |
| rs9941217 | 16 | 18050926 | G | C | 0.3522 | -0.019 | 0.003 | 3.5E-12 | 48.40 |
| rs7188873 | 16 | 24727064 | G | A | 0.613 | 0.020 | 0.003 | 8.46E-15 | 60.20 |
| rs6497840 | 16 | 25351633 | A | G | 0.707 | 0.023 | 0.003 | 2.01E-15 | 63.10 |
| rs4785187 | 16 | 49766772 | A | G | 0.223 | 0.020 | 0.003 | 6.55E-11 | 42.60 |
| rs8050598 | 16 | 49891964 | T | C | 0.2541 | 0.019 | 0.003 | 1.76E-10 | 40.70 |
| rs12918191 | 16 | 50945156 | G | A | 0.243 | -0.020 | 0.003 | 3.14E-11 | 44.10 |
| rs9302604 | 16 | 69576894 | G | A | 0.435 | 0.019 | 0.003 | 3.29E-13 | 53.00 |
| rs9936784 | 16 | 72230694 | G | T | 0.5342 | 0.014 | 0.003 | 4.33E-08 | 30.00 |
| rs62052916 | 16 | 72574550 | T | A | 0.0701 | -0.032 | 0.005 | 1.62E-10 | 40.90 |
| rs4788676 | 16 | 72950468 | C | T | 0.2285 | -0.018 | 0.003 | 4.92E-09 | 34.20 |
| rs61537885 | 16 | 75620118 | C | T | 0.0372 | -0.040 | 0.007 | 8.06E-09 | 33.30 |
| rs117657830 | 16 | 75766873 | G | A | 0.0417 | -0.038 | 0.006 | 3.18E-09 | 35.10 |
| rs1050847 | 16 | 87443734 | T | C | 0.559 | -0.015 | 0.003 | 7.37E-09 | 33.40 |
| rs11642231 | 16 | 89608702 | A | G | 0.369 | -0.016 | 0.003 | 3.44E-09 | 34.90 |
| rs4790874 | 17 | 1995177 | T | C | 0.532 | 0.017 | 0.003 | 8.43E-12 | 46.70 |
| rs11078713 | 17 | 7795972 | G | A | 0.4193 | -0.015 | 0.003 | 1.59E-08 | 31.90 |
| rs28441558 | 17 | 7803118 | C | T | 0.0563 | -0.036 | 0.006 | 1.24E-10 | 41.40 |
| rs11651955 | 17 | 16235462 | A | G | 0.499 | -0.014 | 0.003 | 3.74E-08 | 30.30 |
| rs67777803 | 17 | 27323322 | T | G | 0.172 | -0.025 | 0.003 | 3.18E-13 | 53.10 |
| rs2344976 | 17 | 30685935 | C | T | 0.612 | -0.015 | 0.003 | 7.98E-09 | 33.30 |
| rs3764351 | 17 | 37824339 | A | G | 0.657 | -0.015 | 0.003 | 3.89E-08 | 30.20 |
| rs72836318 | 17 | 44121579 | C | T | 0.246 | -0.017 | 0.003 | 7E-09 | 33.50 |
| rs17692129 | 17 | 44793283 | T | C | 0.331 | 0.020 | 0.003 | 4.57E-13 | 52.40 |
| rs75919030 | 17 | 50193197 | C | T | 0.267 | -0.021 | 0.003 | 3.35E-13 | 53.00 |
| rs2938134 | 17 | 50243397 | A | C | 0.673 | -0.018 | 0.003 | 3.14E-10 | 39.60 |
| rs2587507 | 17 | 77790135 | C | T | 0.502 | -0.015 | 0.003 | 8.69E-09 | 33.10 |
| rs34342129 | 18 | 5872472 | C | T | 0.509 | -0.014 | 0.003 | 2.13E-08 | 31.40 |
| rs4476253 | 18 | 25253297 | A | G | 0.24 | -0.018 | 0.003 | 5.78E-10 | 38.40 |
| rs7505855 | 18 | 31696075 | T | C | 0.586 | -0.017 | 0.003 | 5.31E-11 | 43.10 |
| rs8096225 | 18 | 36921851 | C | A | 0.703 | 0.016 | 0.003 | 2.63E-08 | 31.00 |
| rs67050670 | 18 | 39297254 | G | A | 0.229 | -0.020 | 0.003 | 2.34E-11 | 44.70 |
| rs2359180 | 18 | 41314171 | G | A | 0.369 | -0.014 | 0.003 | 4.98E-08 | 29.70 |
| rs72898831 | 18 | 42658643 | G | A | 0.155 | -0.024 | 0.004 | 4.14E-12 | 48.10 |
| rs8083764 | 18 | 49874515 | T | G | 0.3062 | -0.016 | 0.003 | 7.97E-09 | 33.30 |
| rs1373178 | 18 | 49967811 | G | T | 0.588 | -0.020 | 0.003 | 4.16E-15 | 61.60 |
| rs62098013 | 18 | 50863861 | A | G | 0.3653 | 0.018 | 0.003 | 2.24E-11 | 44.80 |
| rs72938304 | 18 | 53661743 | A | G | 0.113 | -0.027 | 0.004 | 1.36E-11 | 45.70 |
| rs11872397 | 18 | 72535282 | A | G | 0.253 | -0.017 | 0.003 | 5.2E-09 | 34.10 |
| rs71367544 | 18 | 77574374 | T | C | 0.203 | 0.021 | 0.003 | 8.54E-11 | 42.10 |
| rs76608582 | 19 | 4474725 | A | C | 0.0489 | -0.035 | 0.006 | 4.88E-09 | 34.20 |
| rs10853981 | 19 | 4965064 | A | G | 0.3304 | 0.015 | 0.003 | 4.88E-08 | 29.80 |
| rs113230003 | 19 | 18460956 | A | G | 0.255 | -0.019 | 0.003 | 1.05E-10 | 41.70 |
| rs8103660 | 19 | 18566395 | C | T | 0.3544 | 0.016 | 0.003 | 3.03E-09 | 35.20 |
| rs117734003 | 19 | 51129745 | C | G | 0.0673 | 0.030 | 0.005 | 2.57E-09 | 35.50 |
| rs1126757 | 19 | 55879872 | T | C | 0.473 | 0.014 | 0.003 | 2.92E-08 | 30.80 |
| rs6050446 | 20 | 25195509 | G | A | 0.971 | 0.054 | 0.008 | 8.8E-13 | 51.10 |
| rs6058782 | 20 | 29946968 | T | C | 0.908 | 0.030 | 0.004 | 1.78E-11 | 45.20 |
| rs1555445 | 20 | 31175258 | T | A | 0.318 | 0.019 | 0.003 | 7.75E-12 | 46.80 |
| rs6073075 | 20 | 42015801 | A | T | 0.824 | -0.019 | 0.003 | 2.44E-08 | 31.10 |
| rs910912 | 20 | 54462393 | C | T | 0.739 | -0.017 | 0.003 | 7.82E-09 | 33.30 |
| rs6011779 | 20 | 61984317 | T | C | 0.806 | -0.019 | 0.003 | 2.83E-09 | 35.30 |
| rs3810496 | 20 | 62406886 | C | T | 0.6194 | 0.016 | 0.003 | 1.54E-09 | 36.50 |
| rs4818005 | 21 | 40588819 | A | G | 0.581 | -0.020 | 0.003 | 1.09E-14 | 59.70 |
| rs139896 | 22 | 38397797 | C | T | 0.648 | 0.015 | 0.003 | 7.14E-09 | 33.50 |
| rs4822102 | 22 | 42698430 | T | C | 0.618 | -0.017 | 0.003 | 2.78E-10 | 39.80 |
| rs9627272 | 22 | 46442288 | C | G | 0.407 | -0.015 | 0.003 | 2.42E-09 | 35.60 |

SNP, single nucleotide polymorphism; A1, effect allele; A2, other allele; FRQ, effect allele frequency; SE, standard error;

**Supplementary Table 5: Characteristics of genetic instruments associated with coffee consumption.**

| SNP | CHR | BP | A1 | A2 | FRQ | beta | se | P | F |
| --- | --- | --- | --- | --- | --- | --- | --- | --- | --- |
| rs1260326 | 2 | 27730940 | C | C | 0.59 | 0.04 | 0.01 | 1.13E-06 | 16 |
| rs1481012 | 4 | 89039082 | A | A | 0.89 | 0.06 | 0.01 | 1.13E-07 | 36 |
| rs4410790 | 7 | 17284577 | C | C | 0.63 | 0.1 | 0.01 | 1.48E-57 | 100 |
| rs7800944 | 7 | 73035857 | C | C | 0.28 | 0.05 | 0.01 | 7.82E-09 | 25 |
| rs17685 | 7 | 75616105 | A | A | 0.29 | 0.07 | 0.01 | 9.06E-14 | 49 |
| rs6265 | 11 | 27679916 | C | C | 0.81 | 0.04 | 0.01 | 3.4E-07 | 16 |
| rs2472297 | 15 | 75027880 | T | T | 0.24 | 0.14 | 0.01 | 6.89E-44 | 196 |
| rs9902453 | 17 | 28349095 | G | G | 0.46 | 0.03 | 0.01 | 2.26E-06 | 9 |

SNP, single nucleotide polymorphism; A1, effect allele; A2, other allele; FRQ, effect allele frequency; SE, standard error;

**Supplementary Table 6: Characteristics of genetic instruments associated with alcohol consumption.**

| SNP | CHR | BP | A1 | A2 | FRQ | beta | se | P | F |
| --- | --- | --- | --- | --- | --- | --- | --- | --- | --- |
| rs705687 | 1 | 4548453 | G | A | 0.785 | -0.011 | 0.002 | 8.2E-10 | 37.70 |
| rs58107686 | 1 | 33837334 | A | C | 0.328 | -0.010 | 0.002 | 7.8E-10 | 37.80 |
| rs12088813 | 1 | 66407700 | C | A | 0.267 | -0.009 | 0.002 | 1.6E-08 | 32.00 |
| rs5024204 | 1 | 71491890 | T | A | 0.278 | 0.010 | 0.002 | 2.6E-09 | 35.50 |
| rs10753661 | 1 | 165119792 | A | G | 0.684 | -0.009 | 0.002 | 3.8E-08 | 30.30 |
| rs28680958 | 1 | 173848808 | A | G | 0.217 | -0.011 | 0.002 | 5.1E-10 | 38.60 |
| rs823114 | 1 | 205719532 | A | G | 0.553 | 0.009 | 0.001 | 2.3E-09 | 35.70 |
| rs77165542 | 2 | 430975 | T | C | 0.0349 | -0.026 | 0.004 | 5.6E-11 | 42.90 |
| rs1260326 | 2 | 27730940 | C | T | 0.601 | 0.021 | 0.001 | 8.1E-45 | 197.00 |
| rs2178197 | 2 | 27860551 | G | A | 0.56908 | -0.009 | 0.001 | 2.5E-09 | 35.60 |
| rs13383034 | 2 | 45155276 | T | C | 0.329 | 0.015 | 0.002 | 6.3E-22 | 92.60 |
| rs1004787 | 2 | 45159091 | A | G | 0.55092 | 0.008 | 0.001 | 8.4E-09 | 33.20 |
| rs13032049 | 2 | 63581507 | G | A | 0.283 | 0.010 | 0.002 | 3E-10 | 39.70 |
| rs828867 | 2 | 74334462 | A | G | 0.545 | 0.009 | 0.001 | 2.2E-09 | 35.80 |
| rs11692435 | 2 | 98275354 | A | G | 0.0852 | 0.017 | 0.003 | 2.5E-11 | 44.50 |
| rs13024996 | 2 | 144225215 | A | C | 0.364 | -0.011 | 0.002 | 5.7E-13 | 51.90 |
| rs72859280 | 2 | 147956293 | T | G | 0.0362 | 0.023 | 0.004 | 4.4E-09 | 34.40 |
| rs56337305 | 2 | 225475560 | C | T | 0.383 | -0.010 | 0.001 | 1.6E-10 | 40.90 |
| rs13094887 | 3 | 70968431 | T | A | 0.301 | -0.010 | 0.002 | 8.6E-11 | 42.10 |
| rs62250685 | 3 | 85457240 | G | A | 0.614 | -0.014 | 0.002 | 1.1E-21 | 91.60 |
| rs74664784 | 3 | 85475292 | C | T | 0.359 | -0.013 | 0.002 | 1.6E-14 | 59.00 |
| rs13066454 | 3 | 93994255 | T | C | 0.398 | -0.009 | 0.001 | 4.1E-09 | 34.60 |
| rs9838144 | 3 | 131576287 | C | G | 0.209 | -0.010 | 0.002 | 2.7E-08 | 30.90 |
| rs2011092 | 3 | 141124607 | C | T | 0.33864 | -0.009 | 0.002 | 7.4E-09 | 33.40 |
| rs60654199 | 3 | 141267295 | A | C | 0.0629 | -0.017 | 0.003 | 2.9E-08 | 30.80 |
| rs6787172 | 3 | 158187811 | G | T | 0.554 | -0.008 | 0.001 | 4.3E-08 | 30.00 |
| rs3748034 | 4 | 3446091 | T | G | 0.143 | -0.012 | 0.002 | 1.7E-08 | 31.80 |
| rs7682824 | 4 | 39406254 | T | C | 0.548 | 0.008 | 0.002 | 2.8E-08 | 30.90 |
| rs11940694 | 4 | 39414993 | G | A | 0.597 | 0.026 | 0.001 | 3E-68 | 305.00 |
| rs35538052 | 4 | 39418965 | A | G | 0.379 | -0.009 | 0.002 | 1.4E-08 | 32.20 |
| rs4501255 | 4 | 42151306 | G | C | 0.235 | 0.011 | 0.002 | 4.8E-10 | 38.70 |
| rs12499107 | 4 | 99678691 | G | A | 0.1312 | 0.013 | 0.002 | 4.5E-09 | 34.40 |
| rs144198753 | 4 | 99713350 | T | C | 0.01632 | -0.042 | 0.006 | 1.4E-12 | 50.20 |
| rs1154414 | 4 | 100000136 | C | T | 0.14106 | 0.018 | 0.002 | 3.7E-17 | 70.90 |
| rs1229984 | 4 | 100239319 | C | T | 0.963 | 0.151 | 0.004 | <2.2e-308 | 1520.00 |
| rs10028756 | 4 | 100254520 | A | G | 0.12946 | -0.019 | 0.002 | 1.2E-17 | 73.20 |
| rs561222871 | 4 | 100260679 | T | C | 0.04738 | -0.039 | 0.004 | 6.6E-27 | 115.00 |
| rs36052336 | 4 | 100273594 | G | A | 0.0615 | -0.018 | 0.003 | 1.2E-09 | 36.90 |
| rs2165670 | 4 | 100286085 | A | G | 0.10634 | 0.023 | 0.002 | 1.7E-22 | 95.30 |
| rs17029090 | 4 | 100443853 | G | A | 0.0199 | -0.049 | 0.005 | 4.8E-21 | 88.60 |
| rs79139602 | 4 | 100444363 | T | A | 0.02106 | 0.060 | 0.005 | 1.8E-32 | 141.00 |
| rs4699791 | 4 | 101243023 | A | G | 0.09573 | 0.019 | 0.002 | 6.6E-14 | 56.20 |
| rs13107325 | 4 | 103188709 | T | C | 0.0722 | -0.028 | 0.003 | 1.5E-22 | 95.40 |
| rs4690727 | 4 | 143648579 | G | C | 0.718 | 0.011 | 0.002 | 2.4E-11 | 44.60 |
| rs10004020 | 4 | 152968372 | A | G | 0.72 | 0.009 | 0.002 | 2.4E-08 | 31.10 |
| rs12651313 | 4 | 171086393 | G | C | 0.443 | -0.009 | 0.001 | 3.8E-09 | 34.70 |
| rs4916723 | 5 | 87854395 | C | A | 0.416 | -0.010 | 0.001 | 1.7E-11 | 45.30 |
| rs12655091 | 5 | 144412335 | A | G | 0.53 | -0.008 | 0.001 | 1.3E-08 | 32.40 |
| rs55872084 | 5 | 155902003 | T | G | 0.235 | 0.010 | 0.002 | 6.3E-09 | 33.70 |
| rs11739827 | 5 | 166803321 | T | G | 0.451 | -0.008 | 0.001 | 1.2E-08 | 32.50 |
| rs10085696 | 7 | 69783020 | G | A | 0.186 | -0.011 | 0.002 | 1.1E-09 | 37.10 |
| rs6460047 | 7 | 73042443 | C | T | 0.208 | 0.012 | 0.002 | 9.7E-11 | 41.90 |
| rs10236149 | 7 | 98977515 | G | A | 0.123 | -0.013 | 0.002 | 1.2E-09 | 37.00 |
| rs35034355 | 7 | 103840115 | A | G | 0.521 | -0.008 | 0.001 | 2.9E-08 | 30.80 |
| rs6951574 | 7 | 153489744 | C | T | 0.458 | 0.013 | 0.001 | 1.6E-19 | 81.70 |
| rs13250583 | 8 | 20949917 | T | C | 0.213 | -0.010 | 0.002 | 4.7E-08 | 29.80 |
| rs1217091 | 8 | 64527399 | C | T | 0.812 | 0.012 | 0.002 | 7.1E-11 | 42.50 |
| rs28601761 | 8 | 126500031 | G | C | 0.42 | 0.009 | 0.001 | 7.2E-10 | 38.00 |
| rs55932213 | 9 | 108755622 | G | A | 0.73639 | 0.009 | 0.002 | 9.6E-09 | 32.90 |
| rs10978550 | 9 | 109345993 | C | T | 0.206 | -0.012 | 0.002 | 7.2E-11 | 42.50 |
| rs7074871 | 10 | 110507806 | A | G | 0.255 | -0.009 | 0.002 | 1.9E-08 | 31.60 |
| rs17665139 | 10 | 125093880 | T | C | 0.149 | -0.012 | 0.002 | 1.6E-08 | 31.90 |
| rs7950166 | 11 | 8642218 | T | C | 0.637 | -0.010 | 0.002 | 9.9E-11 | 41.80 |
| rs11030084 | 11 | 27643725 | T | C | 0.184 | -0.011 | 0.002 | 1.7E-08 | 31.80 |
| rs56030824 | 11 | 47397353 | A | G | 0.322 | -0.012 | 0.002 | 1.2E-13 | 55.10 |
| rs10750025 | 11 | 113424042 | T | C | 0.686 | 0.010 | 0.002 | 4.9E-11 | 43.20 |
| rs1713676 | 11 | 113660576 | G | A | 0.52245 | -0.008 | 0.001 | 4.3E-08 | 30.00 |
| rs4938230 | 11 | 116075001 | A | C | 0.842 | 0.013 | 0.002 | 1.5E-10 | 41.10 |
| rs682011 | 11 | 121544285 | C | T | 0.559 | 0.008 | 0.001 | 2.2E-08 | 31.30 |
| rs12795042 | 11 | 133658168 | C | A | 0.623 | -0.008 | 0.002 | 3.3E-08 | 30.60 |
| rs10876188 | 12 | 51895882 | T | C | 0.457 | -0.008 | 0.001 | 4.8E-08 | 29.80 |
| rs3809162 | 12 | 54674235 | G | A | 0.397 | 0.009 | 0.001 | 1.2E-09 | 37.00 |
| rs10506274 | 12 | 81601464 | T | G | 0.484 | -0.009 | 0.001 | 5.8E-10 | 38.40 |
| rs4842786 | 12 | 92170791 | A | G | 0.584 | -0.009 | 0.001 | 2.7E-09 | 35.40 |
| rs500321 | 13 | 27124360 | T | A | 0.736 | -0.010 | 0.002 | 4.9E-09 | 34.20 |
| rs1123285 | 14 | 57274519 | G | C | 0.335 | -0.009 | 0.002 | 8.1E-09 | 33.20 |
| rs2180870 | 14 | 58782779 | C | T | 0.135 | -0.012 | 0.002 | 1.1E-08 | 32.60 |
| rs28929474 | 14 | 94844947 | T | C | 0.0183 | -0.037 | 0.005 | 1.3E-11 | 45.80 |
| rs11625650 | 14 | 104610138 | A | G | 0.233 | -0.010 | 0.002 | 2.9E-08 | 30.80 |
| rs2472297 | 15 | 75027880 | T | C | 0.249 | 0.011 | 0.002 | 3.1E-10 | 39.60 |
| rs12907323 | 15 | 86796012 | G | A | 0.411 | 0.008 | 0.001 | 9.9E-09 | 32.90 |
| rs2764771 | 16 | 20013793 | A | G | 0.307 | 0.010 | 0.002 | 4E-10 | 39.10 |
| rs17177078 | 16 | 24810681 | T | C | 0.0626 | -0.022 | 0.003 | 1.3E-13 | 54.90 |
| rs378421 | 16 | 28754684 | A | G | 0.404 | -0.011 | 0.001 | 4.8E-14 | 56.80 |
| rs113443718 | 16 | 29892184 | A | G | 0.305 | -0.010 | 0.002 | 1.2E-10 | 41.50 |
| rs62044525 | 16 | 64872590 | G | C | 0.184 | -0.012 | 0.002 | 1E-10 | 41.80 |
| rs7185555 | 16 | 69131281 | C | G | 0.153 | -0.011 | 0.002 | 4.2E-08 | 30.00 |
| rs79616692 | 16 | 72338507 | C | G | 0.108 | 0.016 | 0.002 | 4.1E-12 | 48.10 |
| rs1104608 | 16 | 73912588 | C | G | 0.425 | -0.011 | 0.001 | 1.1E-13 | 55.30 |
| rs4548913 | 17 | 2209888 | A | G | 0.632 | -0.008 | 0.002 | 3.1E-08 | 30.60 |
| rs3803800 | 17 | 7462969 | G | A | 0.786 | 0.011 | 0.002 | 1.5E-10 | 41.00 |
| rs2854334 | 17 | 29715500 | G | A | 0.615 | 0.009 | 0.001 | 7.5E-10 | 37.90 |
| rs2532276 | 17 | 44246624 | A | C | 0.215 | -0.022 | 0.003 | 1.6E-17 | 72.60 |
| rs10438820 | 17 | 78524597 | T | C | 0.702 | 0.009 | 0.002 | 1.8E-08 | 31.70 |
| rs9950000 | 18 | 53052169 | T | C | 0.395 | -0.009 | 0.001 | 9.4E-10 | 37.40 |
| rs4092465 | 18 | 55080437 | G | A | 0.635 | -0.008 | 0.002 | 4.4E-08 | 30.00 |
| rs281379 | 19 | 49214274 | A | G | 0.508 | 0.014 | 0.001 | 4.9E-21 | 88.60 |
| rs4815364 | 20 | 25035711 | A | G | 0.616 | 0.009 | 0.001 | 1E-08 | 32.80 |
| rs9607814 | 22 | 41946519 | A | C | 0.2 | -0.010 | 0.002 | 4.3E-08 | 30.00 |

SNP, single nucleotide polymorphism; A1, effect allele; A2, other allele; FRQ, effect allele frequency; SE, standard error;

**Supplementary Table 7. The heterogeneity test of the genetic instruments**

| **Exposure** | **Outcome** | **Q** | **Q_pval** |
| --- | --- | --- | --- |
| CoffeeConsumption | cloz | 12.19376899 | 0.094364049 |
| SmokingCessation | cloz | 14.21731681 | 0.433649488 |
| AgeofSmoking | cloz | 5.070809064 | 0.749981853 |
| SmokingInitiation | cloz | 233.4608336 | 0.047496956 |
| CPD | cloz | 31.4837296 | 0.724951969 |
| DrinksPerWeek | cloz | 137.2790983 | 4.01E-06 |
| CoffeeConsumption | norcloz | 10.76605305 | 0.149154218 |
| SmokingCessation | norcloz | 16.49035534 | 0.284354435 |
| AgeofSmoking | norcloz | 2.678445902 | 0.952890112 |
| SmokingInitiation | norcloz | 226.047971 | 0.091449649 |
| CPD | norcloz | 32.8500329 | 0.663997624 |
| DrinksPerWeek | norcloz | 111.2113777 | 0.001623429 |
| CoffeeConsumption | ratio | 13.22877475 | 0.066726675 |
| SmokingCessation | ratio | 13.00106148 | 0.526440042 |
| AgeofSmoking | ratio | 4.370111688 | 0.822282983 |
| SmokingInitiation | ratio | 257.4955737 | 0.003278811 |
| CPD | ratio | 25.25056608 | 0.928562174 |
| DrinksPerWeek | ratio | 94.30205739 | 0.033655618 |

**Supplementary Table 8. The directional pleiotropy in the MR-Egger analysis.**

| **exposure** | **outcome** | **egger_intercept** | **se** | **pval** |
| --- | --- | --- | --- | --- |
| CoffeeConsumption | cloz | 0.0162 | 0.0106 | 0.18 |
| SmokingCessation | cloz | 0.0052 | 0.0091 | 0.57 |
| AgeofSmoking | cloz | 0.0077 | 0.0229 | 0.75 |
| SmokingInitiation | cloz | 0.0057 | 0.0036 | 0.11 |
| CPD | cloz | 0.0010 | 0.0033 | 0.76 |
| DrinksPerWeek | cloz | -0.0008 | 0.0039 | 0.83 |
| CoffeeConsumption | norcloz | 0.0162 | 0.0089 | 0.12 |
| SmokingCessation | norcloz | 0.0084 | 0.0090 | 0.37 |
| AgeofSmoking | norcloz | 0.0147 | 0.0216 | 0.52 |
| SmokingInitiation | norcloz | 0.0030 | 0.0033 | 0.37 |
| CPD | norcloz | 0.0000 | 0.0031 | 0.99 |
| DrinksPerWeek | norcloz | 0.0001 | 0.0033 | 0.97 |
| CoffeeConsumption | ratio | 0.0008 | 0.0072 | 0.92 |
| SmokingCessation | ratio | -0.0025 | 0.0049 | 0.62 |
| AgeofSmoking | ratio | -0.0058 | 0.0127 | 0.66 |
| SmokingInitiation | ratio | 0.0024 | 0.0021 | 0.25 |
| CPD | ratio | 0.0010 | 0.0018 | 0.57 |
| DrinksPerWeek | ratio | -0.0008 | 0.0018 | 0.65 |

# Supplemental Figures

**Figure S1: Plot from leave-one-out sensitivity analysis for the instrument variable set.** The solid lines represent 95% confidence intervals. Leaving-one-out analysis revealed that no single SNP had a significant influence on the results of clozapine for smoking initiation.


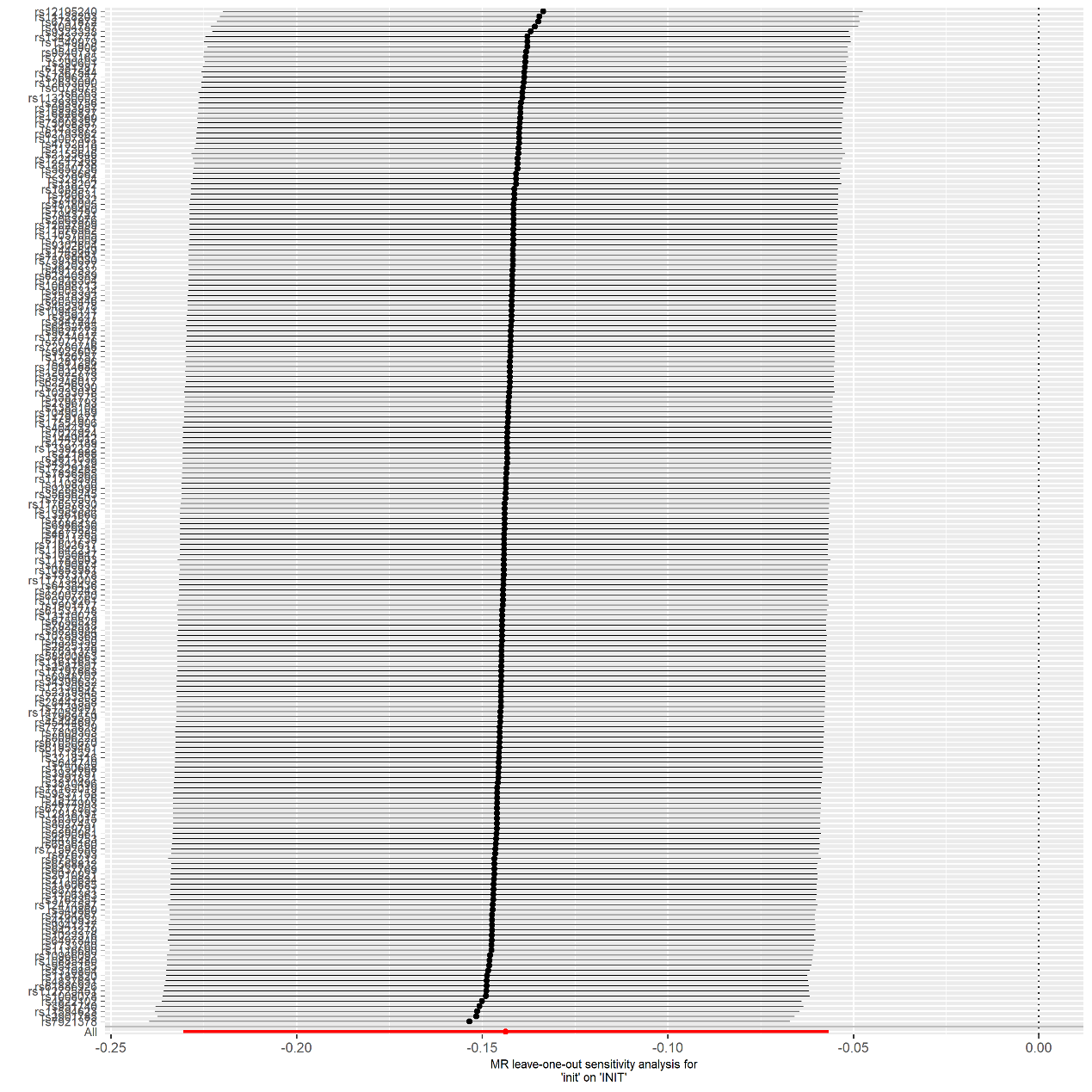


**Figure S2: Plot from leave-one-out sensitivity analysis for the instrument variable set.** The solid lines represent 95% confidence intervals. Leaving-one-out analysis revealed that no single SNP had a significant influence on the results of clozapine for coffee consumption


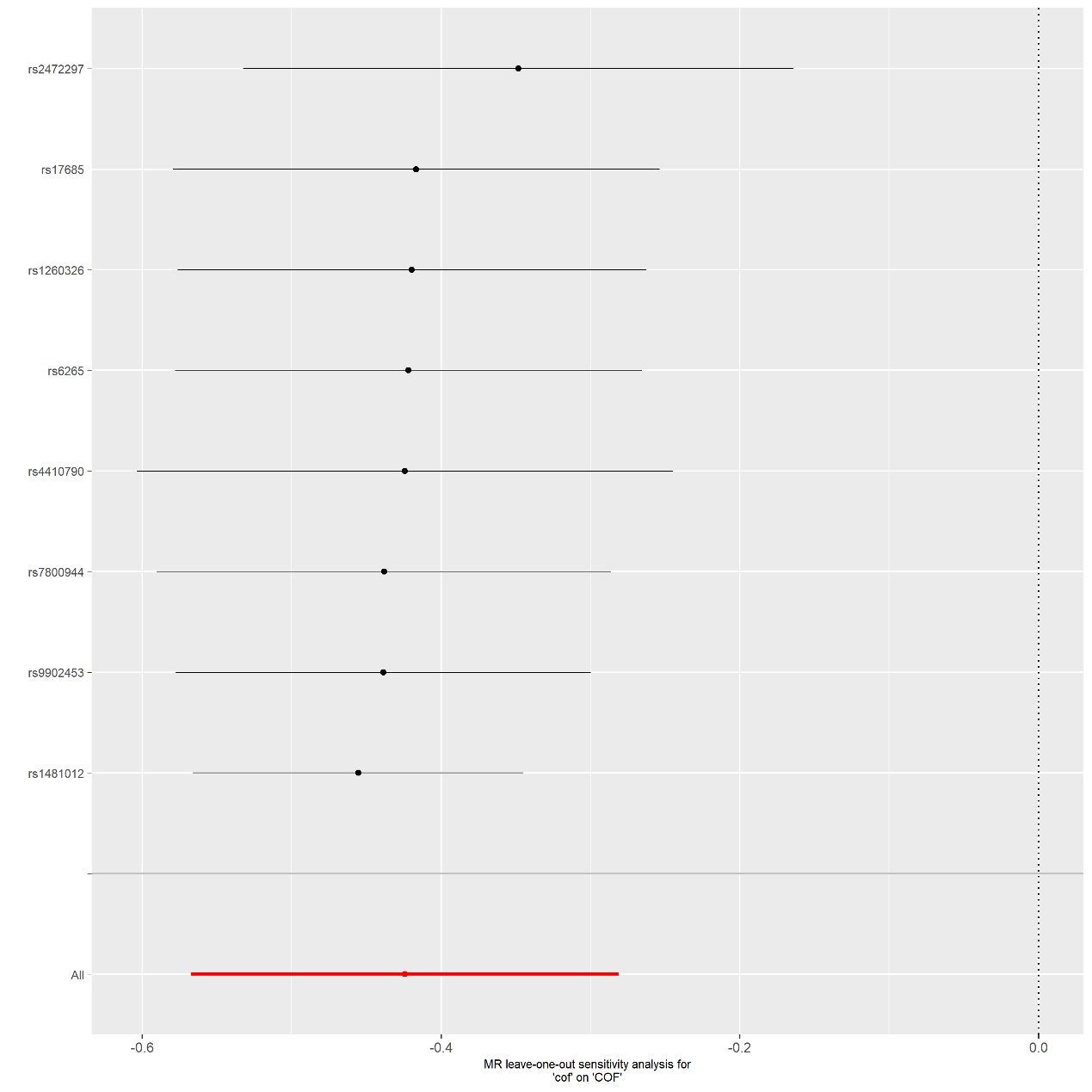


**Figure S3: Plot from leave-one-out sensitivity analysis for the instrument variable set.** The solid lines represent 95% confidence intervals. Leaving-one-out analysis revealed that no single SNP had a significant influence on the results of norclozapine for smoking initiation.


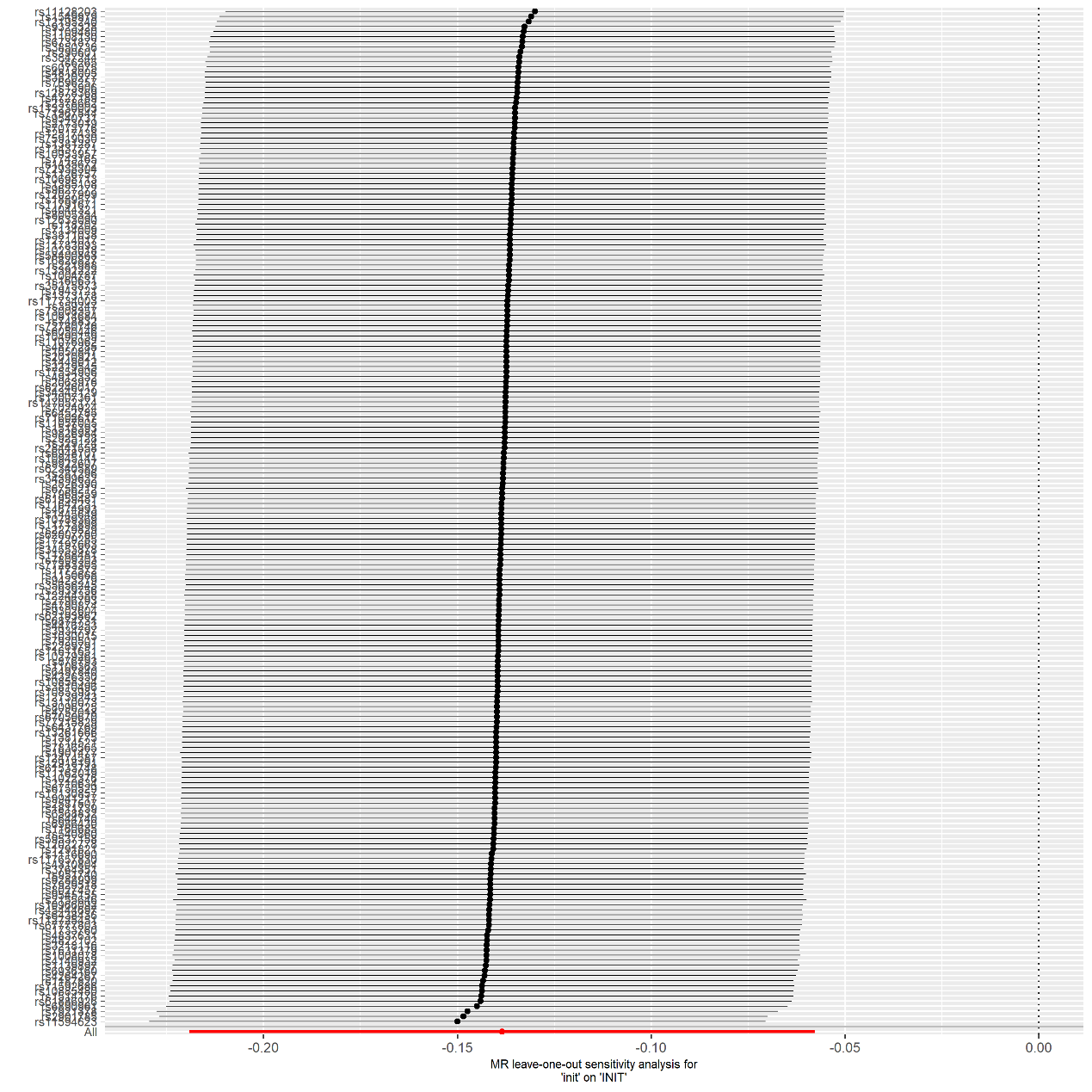


**Figure S4:** **Plot from leave-one-out sensitivity analysis for the instrument variable set.** The solid lines represent 95% confidence intervals. leaving-one-out analysis revealed that no single SNP had a significant influence on the results of norclozapine for coffee consumption.


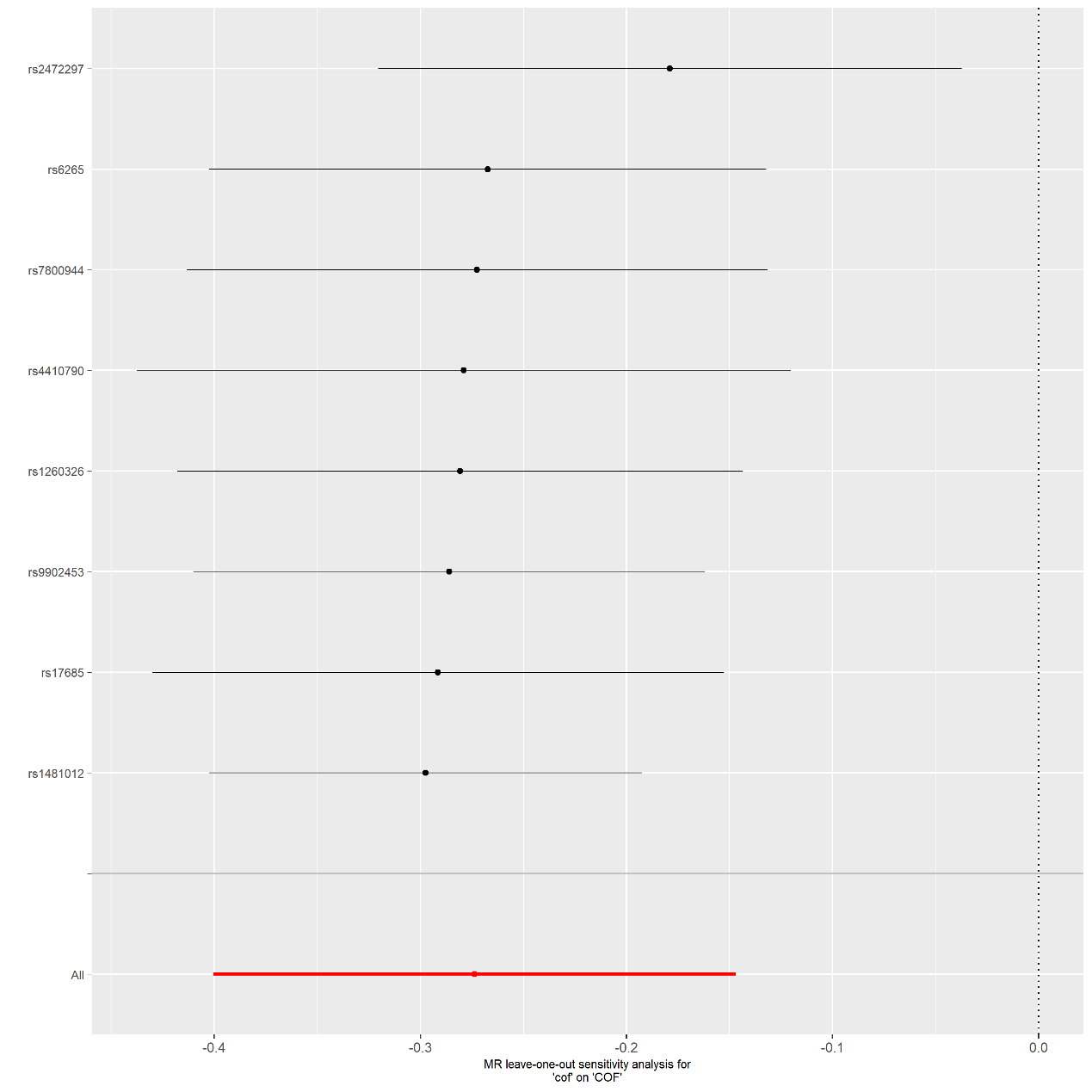


**Figure S5: Plot from leave-one-out sensitivity analysis for the instrument variable set.** The solid lines represent 95% confidence intervals. leaving-one-out analysis revealed that no single SNP had a significant influence on the results of the metabolic ratio for coffee consumption.


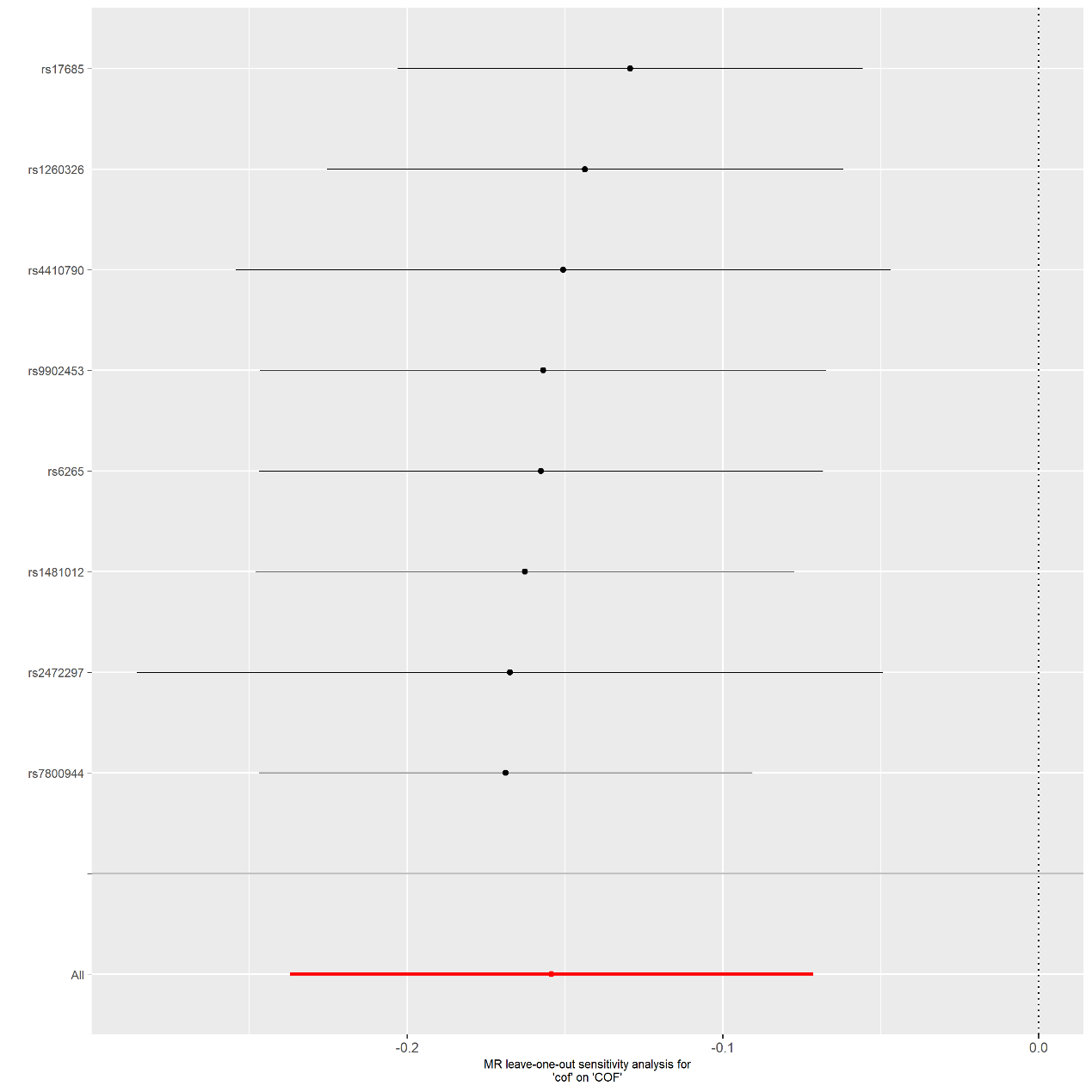

Supplement: Supplementary file 1 [file Data_Sheet_1.docx]
